# Supplementary material for: Medfly Population Suppression through Augmentative Release of an Introduced Parasitoid in an Irrigated Multi-Fruit Orchard of Central–Western Argentina
Source: Insects. 2023 Apr 16;14(4):387. doi: 10.3390/insects14040387 (PMC10140834; doi:10.3390/insects14040387)
Supplement: Supplementary file 1 [file insects-14-00387-s001.zip › File S10_xmedfly.pdf]

season	treatment	idrelease	idtrial	pupaC. mean	adultC .mean	adult D.mean	nopupa C.mean	emerg C.mean	emerg D.mean	pupa C.sd	adult C.sd	adult D.sd	nopupa C.sd	emerg C.sd	emerg D.sd	pupaC. median	adultC. median	adultD. median	nopupaC .median	emergC. median	emergD. median	ftd. mean	femrelease. mean	xtemp. mean	xhr. mean	xprec. mean	prec. acum
2013	non release farm	1	1	20,10	12,40	0,00	7,70	0,62	0,00	4,72	3,17	0,00	2,58	0,07	0,00	20,50	12,00	0,00	7,50	0,59	0,00	1,00	0,00	25,89	37,92	0,75	10,00
2013	non release farm	2	2	23,70	13,40	0,00	10,30	0,57	0,00	8,04	4,40	0,00	3,86	0,04	0,00	21,50	12,00	0,00	10,00	0,56	0,00	1,19	0,00	27,99	46,79	0,35	4,00
2013	non release farm	3	3	18,20	10,50	0,00	7,70	0,57	0,00	7,66	5,06	0,00	3,13	0,07	0,00	16,00	9,00	0,00	6,50	0,56	0,00	1,08	0,00	25,79	62,83	3,85	46,00
2013	non release farm	4	4	20,30	12,80	0,00	7,50	0,64	0,00	5,21	3,65	0,00	3,27	0,11	0,00	19,00	11,50	0,00	7,50	0,62	0,00	1,15	0,00	27,13	51,58	0,00	0,50
2013	non release farm	5	5	24,10	14,40	0,00	9,70	0,60	0,00	4,75	2,22	0,00	2,67	0,04	0,00	24,00	14,50	0,00	9,50	0,60	0,00	1,06	0,00	26,76	47,92	1,50	16,00
2013	non release farm	6	6	26,70	15,30	0,00	10,40	0,58	0,00	4,90	3,62	0,00	2,80	0,10	0,00	26,50	15,00	0,00	11,00	0,58	0,00	1,00	0,00	23,75	47,46	1,23	16,00
2013	non release farm	7	7	30,50	19,10	0,00	11,40	0,63	0,00	6,00	3,48	0,00	3,27	0,06	0,00	30,00	19,50	0,00	11,50	0,62	0,00	1,17	0,00	20,98	55,17	0,03	1,00
2013	non release farm	8	8	26,80	16,80	0,00	10,00	0,62	0,00	8,85	6,71	0,00	3,27	0,10	0,00	24,50	14,00	0,00	9,50	0,62	0,00	0,92	0,00	20,10	56,45	0,45	5,00
2013	non release farm	9	9	23,90	13,60	0,00	10,30	0,57	0,00	6,79	4,35	0,00	3,16	0,07	0,00	23,50	13,50	0,00	11,00	0,55	0,00	1,00	0,00	18,64	57,85	0,07	0,50
2013	non release farm	10	10	15,60	8,20	0,00	7,40	0,52	0,00	4,27	2,82	0,00	1,78	0,07	0,00	14,50	8,00	0,00	7,50	0,53	0,00	0,85	0,00	20,00	57,35	0,08	1,00
2013	non release farm	11	11	11,80	6,00	0,00	5,80	0,50	0,00	3,71	3,30	0,00	2,53	0,15	0,00	12,50	5,00	0,00	5,00	0,52	0,00	0,96	0,00	18,00	65,00	0,05	1,00
2013	non release farm	12	12	14,00	6,70	0,00	7,30	0,48	0,00	3,97	2,91	0,00	3,59	0,18	0,00	13,00	7,00	0,00	5,50	0,48	0,00	0,81	0,00	16,00	61,00	0,08	1,00
2013	non release farm	13	13	13,10	6,60	0,00	6,50	0,49	0,00	5,36	4,55	0,00	3,57	0,24	0,00	11,50	5,50	0,00	5,50	0,59	0,00	0,47	0,00	14,00	62,00	0,07	1,00
2013	release farm	1	1	22,80	9,60	2,90	10,30	0,42	0,14	5,96	2,80	0,99	3,92	0,06	0,06	23,00	8,50	3,00	9,50	0,43	0,13	1,10	8.400,00	26,11	37,92	0,75	9,00
2013	release farm	2	2	19,90	8,50	2,40	9,00	0,42	0,13	5,69	2,95	1,35	2,83	0,09	0,08	20,50	10,00	2,50	8,00	0,41	0,13	1,07	8.520,00	27,99	46,79	0,35	4,20
2013	release farm	3	3	21,60	7,70	3,00	10,90	0,34	0,15	5,74	3,16	1,15	2,77	0,08	0,09	22,00	8,00	3,00	12,00	0,37	0,12	1,11	8.700,00	25,79	62,83	3,85	46,20
2013	release farm	4	4	22,20	6,70	3,80	11,30	0,29	0,19	6,66	3,16	2,10	4,52	0,08	0,11	22,00	7,00	3,50	10,50	0,29	0,19	0,96	9.000,00	27,13	51,58	0,00	0,00
2013	release farm	5	5	23,60	5,10	2,30	16,20	0,21	0,10	7,78	2,96	1,34	5,92	0,10	0,05	24,00	4,50	2,50	14,50	0,20	0,09	0,73	9.450,00	26,76	47,92	1,50	18,00
2013	release farm	6	6	22,10	7,00	6,00	9,20	0,31	0,27	4,98	2,31	2,40	2,30	0,06	0,08	21,50	6,50	6,00	9,00	0,32	0,28	0,92	8.970,00	23,75	47,46	1,23	16,00
2013	release farm	7	7	17,50	4,90	3,10	9,70	0,27	0,18	7,14	3,21	1,45	3,53	0,12	0,06	18,00	4,00	3,00	8,50	0,29	0,17	0,79	9.300,00	20,98	55,17	0,03	0,30
2013	release farm	8	8	15,80	3,20	3,00	9,60	0,17	0,19	5,67	3,16	1,33	3,13	0,15	0,08	14,00	3,00	3,00	10,50	0,22	0,17	0,52	9.000,00	20,10	58,42	0,46	5,50
2013	release farm	9	9	12,10	1,10	3,10	7,90	0,09	0,25	1,97	0,74	1,29	1,45	0,06	0,08	12,50	1,00	3,50	7,50	0,10	0,25	0,52	9.000,00	18,89	58,75	0,07	1,00
2013	release farm	10	10	9,20	1,40	1,90	5,90	0,11	0,22	3,71	2,01	0,99	1,79	0,14	0,13	8,50	0,50	1,50	6,50	0,05	0,18	0,46	9.000,00	19,43	55,67	0,08	1,00
2013	release farm	11	11	8,30	0,60	2,00	5,70	0,07	0,22	2,00	0,70	1,49	0,95	0,08	0,15	8,50	0,50	2,00	6,00	0,06	0,21	0,42	8.610,00	17,75	63,58	0,06	0,70
2013	release farm	12	12	5,40	0,60	0,90	3,90	0,08	0,16	2,67	0,70	0,57	1,91	0,09	0,11	6,00	0,50	1,00	4,00	0,07	0,15	0,29	8.700,00	15,40	56,79	0,08	1,00
2013	release farm	13	13	3,60	0,30	0,80	2,50	0,05	0,20	2,50	0,48	0,63	1,72	0,08	0,16	4,00	0,00	1,00	3,00	0,00	0,23	0,19	8.550,00	12,76	60,64	0,07	1,00
2014	non release farm	1	14	26,30	14,80	0,00	11,50	0,55	0,00	5,06	4,69	0,00	2,27	0,09	0,00	25,00	13,50	0,00	10,50	0,57	0,00	1,38	0,00	29,58	50,96	2,00	24,00
2014	non release farm	2	15	24,20	14,90	0,00	9,30	0,62	0,00	9,48	5,57	0,00	4,11	0,05	0,00	22,00	13,50	0,00	8,50	0,61	0,00	1,26	0,00	27,75	47,25	0,63	8,00
2014	non release farm	3	16	25,00	15,10	0,00	9,90	0,60	0,00	7,27	5,22	0,00	2,69	0,06	0,00	24,00	14,50	0,00	9,00	0,61	0,00	1,23	0,00	29,18	47,92	3,81	43,00
2014	non release farm	4	17	27,10	15,60	0,00	11,50	0,57	0,00	6,98	4,95	0,00	3,34	0,08	0,00	26,00	15,50	0,00	11,50	0,59	0,00	1,10	0,00	26,71	47,54	0,51	5,90
2014	non release farm	5	18	21,00	11,50	0,00	9,20	0,54	0,00	6,34	3,89	0,00	2,35	0,05	0,00	21,00	12,00	0,00	9,00	0,57	0,00	1,31	0,00	26,63	51,71	2,13	25,60
2014	non release farm	6	19	23,60	14,90	0,00	8,70	0,62	0,00	6,36	5,15	0,00	2,54	0,11	0,00	24,00	15,00	0,00	8,50	0,64	0,00	1,04	0,00	29,11	55,19	1,92	25,00
2014	non release farm	7	20	20,40	11,30	0,00	9,10	0,57	0,00	4,35	2,11	0,00	4,01	0,11	0,00	20,50	10,50	0,00	8,50	0,56	0,00	1,15	0,00	23,08	53,42	0,21	3,00
2014	non release farm	8	21	22,60	15,20	0,00	7,20	0,66	0,00	5,62	5,07	0,00	1,62	0,09	0,00	21,50	15,00	0,00	7,00	0,66	0,00	1,27	0,00	22,00	56,20	0,12	2,00
2014	non release farm	9	22	24,00	15,10	0,00	8,90	0,63	0,00	8,89	5,43	0,00	3,87	0,06	0,00	21,50	13,50	0,00	8,50	0,64	0,00	1,10	0,00	20,00	64,35	0,38	4,00
2014	non release farm	10	23	21,40	11,70	0,00	9,70	0,56	0,00	6,20	4,52	0,00	5,25	0,16	0,00	20,50	11,50	0,00	7,50	0,60	0,00	1,08	0,00	18,00	68,00	0,23	3,00
2014	non release farm	11	24	16,90	9,00	0,00	7,90	0,53	0,00	3,87	4,16	0,00	3,96	0,20	0,00	16,50	9,00	0,00	7,00	0,67	0,00	1,00	0,00	17,00	63,00	0,25	3,00
2014	non release farm	12	25	16,30	8,60	0,00	7,70	0,50	0,00	5,38	4,43	0,00	2,36	0,18	0,00	15,00	9,00	0,00	7,00	0,57	0,00	0,85	0,00	16,00	67,00	0,08	1,50

2014	non release farm	13	26	15,50	7,50	0,00	8,00	0,48	0,00	2,99	3,17	0,00	3,02	0,18	0,00	15,00	6,50	0,00	8,50	0,42	0,00	0,60	0,00	10,00	58,00	0,14	1,50
2014	release farm	1	14	18,70	7,40	3,50	7,80	0,40	0,18	6,00	2,63	2,01	2,74	0,09	0,06	17,50	6,50	3,00	7,50	0,41	0,17	0,90	8.580,00	29,58	50,95	2,00	24,10
2014	release farm	2	15	21,10	7,50	5,20	8,40	0,35	0,25	3,57	2,84	2,49	2,76	0,10	0,13	22,50	6,50	4,50	7,50	0,32	0,22	1,07	8.550,00	28,79	47,13	0,60	7,60
2014	release farm	3	16	20,20	6,20	5,30	8,70	0,30	0,26	3,36	2,44	1,95	1,34	0,10	0,06	20,00	6,50	5,00	8,50	0,32	0,26	0,78	9.000,00	29,19	47,92	3,81	41,90
2014	release farm	4	17	14,60	4,40	4,40	5,80	0,30	0,29	3,60	1,43	2,17	1,23	0,07	0,08	14,00	4,50	3,50	6,00	0,31	0,29	0,73	8.700,00	26,70	47,54	0,51	6,10
2014	release farm	5	18	13,90	4,40	3,80	5,70	0,31	0,28	4,04	2,07	1,48	1,64	0,09	0,07	13,50	4,50	4,00	6,00	0,31	0,28	0,63	9.000,00	26,63	51,71	2,10	25,60
2014	release farm	6	19	14,10	4,10	3,70	6,30	0,28	0,26	3,73	1,79	1,49	1,42	0,08	0,07	14,00	4,00	3,50	6,00	0,28	0,29	0,50	8.940,00	29,11	55,19	1,92	25,00
2014	release farm	7	20	11,60	2,20	3,50	5,90	0,19	0,31	2,17	0,92	0,97	1,73	0,07	0,08	11,00	2,00	3,00	6,00	0,17	0,30	0,58	9.150,00	23,08	53,42	0,21	2,50
2014	release farm	8	21	13,00	2,90	4,10	6,30	0,21	0,33	3,56	2,02	1,45	2,21	0,11	0,13	12,50	2,00	4,00	5,50	0,19	0,34	0,54	9.000,00	22,31	56,92	0,13	1,50
2014	release farm	9	22	11,40	2,10	4,00	5,30	0,17	0,35	2,76	1,45	1,15	1,25	0,08	0,08	10,50	1,50	3,50	5,50	0,15	0,33	0,40	8.970,00	19,77	63,82	0,39	5,40
2014	release farm	10	23	10,50	0,70	3,20	6,60	0,06	0,31	1,72	1,06	1,03	1,35	0,09	0,10	10,50	0,00	3,00	6,50	0,00	0,31	0,35	9.000,00	17,42	66,17	0,23	2,80
2014	release farm	11	24	6,90	0,40	2,00	4,50	0,05	0,26	3,18	0,52	1,25	2,12	0,07	0,14	7,00	0,00	2,00	5,00	0,00	0,29	0,50	9.000,00	16,33	60,83	0,26	3,10
2014	release farm	12	25	6,00	0,20	1,80	4,00	0,03	0,24	3,40	0,42	1,23	2,31	0,06	0,14	7,00	0,00	2,00	5,00	0,00	0,25	0,21	8.400,00	15,49	68,17	0,08	1,00
2014	release farm	13	26	4,60	0,10	1,30	3,20	0,01	0,20	3,63	0,32	1,06	2,53	0,04	0,14	5,00	0,00	1,50	3,50	0,00	0,25	0,08	8.460,00	11,20	60,11	0,14	2,00
