# Supplementary material for: Medfly Population Suppression through Augmentative Release of an Introduced Parasitoid in an Irrigated Multi-Fruit Orchard of Central–Western Argentina
Source: Insects. 2023 Apr 16;14(4):387. doi: 10.3390/insects14040387 (PMC10140834; doi:10.3390/insects14040387)
Supplement: Supplementary file 1 [file insects-14-00387-s001.zip › File S8_fullmedfly.pdf]

season	odate	pdate	ddate	idrelease	iddevice	idd	puparia	medfly adults	parasitoids	unemerged puparia	medfly emergence	parasitism	medfly mortality	puparia mortality	treatment
2013	21-12-12	26-12-12	02-01-13	1	D1	d1	30	13	3	14	0,43	0,10	0,57	0,47	release farm
2013	21-12-12	26-12-12	02-01-13	1	D2	d2	17	8	2	7	0,47	0,12	0,53	0,41	release farm
2013	21-12-12	26-12-12	02-01-13	1	D3	d3	31	14	2	15	0,45	0,06	0,55	0,48	release farm
2013	21-12-12	26-12-12	02-01-13	1	D4	d4	24	10	5	9	0,42	0,21	0,58	0,38	release farm
2013	21-12-12	26-12-12	02-01-13	1	D5	d5	29	9	3	17	0,31	0,10	0,69	0,59	release farm
2013	21-12-12	26-12-12	02-01-13	1	D6	d6	22	8	3	11	0,36	0,14	0,64	0,50	release farm
2013	21-12-12	26-12-12	02-01-13	1	D7	d7	16	7	4	5	0,44	0,25	0,56	0,31	release farm
2013	21-12-12	26-12-12	02-01-13	1	D8	d8	25	13	2	10	0,52	0,08	0,48	0,40	release farm
2013	21-12-12	26-12-12	02-01-13	1	D9	d9	19	8	3	8	0,42	0,16	0,58	0,42	release farm
2013	21-12-12	26-12-12	02-01-13	1	D10	d10	15	6	2	7	0,40	0,13	0,60	0,47	release farm
2013	02-01-13	07-01-13	14-01-13	2	D1	d11	12	4	4	4	0,33	0,33	0,67	0,33	release farm
2013	02-01-13	07-01-13	14-01-13	2	D2	d12	21	10	3	8	0,48	0,14	0,52	0,38	release farm
2013	02-01-13	07-01-13	14-01-13	2	D3	d13	27	11	4	12	0,41	0,15	0,59	0,44	release farm
2013	02-01-13	07-01-13	14-01-13	2	D4	d14	22	10	1	11	0,45	0,05	0,55	0,50	release farm
2013	02-01-13	07-01-13	14-01-13	2	D5	d15	29	11	4	14	0,38	0,14	0,62	0,48	release farm
2013	02-01-13	07-01-13	14-01-13	2	D6	d16	21	8	3	10	0,38	0,14	0,62	0,48	release farm
2013	02-01-13	07-01-13	14-01-13	2	D7	d17	17	7	2	8	0,41	0,12	0,59	0,47	release farm
2013	02-01-13	07-01-13	14-01-13	2	D8	d18	20	11	1	8	0,55	0,05	0,45	0,40	release farm
2013	02-01-13	07-01-13	14-01-13	2	D9	d19	19	10	1	8	0,53	0,05	0,47	0,42	release farm
2013	02-01-13	07-01-13	14-01-13	2	D10	d20	11	3	1	7	0,27	0,09	0,73	0,64	release farm
2013	14-01-13	19-01-13	26-01-13	3	D1	d21	20	8	1	11	0,40	0,05	0,60	0,55	release farm
2013	14-01-13	19-01-13	26-01-13	3	D2	d22	27	10	3	14	0,37	0,11	0,63	0,52	release farm
2013	14-01-13	19-01-13	26-01-13	3	D3	d23	24	8	3	13	0,33	0,13	0,67	0,54	release farm
2013	14-01-13	19-01-13	26-01-13	3	D4	d24	18	5	4	9	0,28	0,22	0,72	0,50	release farm
2013	14-01-13	19-01-13	26-01-13	3	D5	d25	27	12	3	12	0,44	0,11	0,56	0,44	release farm
2013	14-01-13	19-01-13	26-01-13	3	D6	d26	29	11	5	13	0,38	0,17	0,62	0,45	release farm
2013	14-01-13	19-01-13	26-01-13	3	D7	d27	16	6	2	8	0,38	0,13	0,63	0,50	release farm
2013	14-01-13	19-01-13	26-01-13	3	D8	d28	19	5	2	12	0,26	0,11	0,74	0,63	release farm
2013	14-01-13	19-01-13	26-01-13	3	D9	d29	25	10	3	12	0,40	0,12	0,60	0,48	release farm
2013	14-01-13	19-01-13	26-01-13	3	D10	d30	11	2	4	5	0,18	0,36	0,82	0,45	release farm
2013	26-01-13	31-01-13	07-02-13	4	D1	d31	23	8	4	11	0,35	0,17	0,65	0,48	release farm
2013	26-01-13	31-01-13	07-02-13	4	D2	d32	33	10	2	21	0,30	0,06	0,70	0,64	release farm
2013	26-01-13	31-01-13	07-02-13	4	D3	d33	25	11	2	12	0,44	0,08	0,56	0,48	release farm
2013	26-01-13	31-01-13	07-02-13	4	D4	d34	32	10	7	15	0,31	0,22	0,69	0,47	release farm
2013	26-01-13	31-01-13	07-02-13	4	D5	d35	23	8	1	14	0,35	0,04	0,65	0,61	release farm
2013	26-01-13	31-01-13	07-02-13	4	D6	d36	21	6	6	9	0,29	0,29	0,71	0,43	release farm
2013	26-01-13	31-01-13	07-02-13	4	D7	d37	16	2	2	8	0,13	0,13	0,63	0,50	release farm
2013	26-01-13	31-01-13	07-02-13	4	D8	d38	13	3	5	5	0,23	0,38	0,77	0,38	release farm

2013	26-01-13	31-01-13	07-02-13	4	D9	d39	15	4	3	8	0,27	0,20	0,73	0,53	release farm
2013	26-01-13	31-01-13	07-02-13	4	D10	d40	21	5	6	10	0,24	0,29	0,76	0,48	release farm
2013	07-02-13	12-02-13	19-02-13	5	D1	d41	17	4	3	10	0,24	0,18	0,76	0,59	release farm
2013	07-02-13	12-02-13	19-02-13	5	D2	d42	32	9	2	21	0,28	0,06	0,72	0,66	release farm
2013	07-02-13	12-02-13	19-02-13	5	D3	d43	31	5	3	23	0,16	0,10	0,84	0,74	release farm
2013	07-02-13	12-02-13	19-02-13	5	D4	d44	34	6	5	23	0,18	0,15	0,82	0,68	release farm
2013	07-02-13	12-02-13	19-02-13	5	D5	d45	26	11	3	12	0,42	0,12	0,58	0,46	release farm
2013	07-02-13	12-02-13	19-02-13	5	D6	d46	28	3	1	24	0,11	0,04	0,89	0,86	release farm
2013	07-02-13	12-02-13	19-02-13	5	D7	d47	22	4	3	15	0,18	0,14	0,82	0,68	release farm
2013	07-02-13	12-02-13	19-02-13	5	D8	d48	20	5	1	14	0,25	0,05	0,75	0,70	release farm
2013	07-02-13	12-02-13	19-02-13	5	D9	d49	12	1	1	10	0,08	0,08	0,92	0,83	release farm
2013	07-02-13	12-02-13	19-02-13	5	D10	d50	14	3	1	10	0,21	0,07	0,79	0,71	release farm
2013	19-02-13	25-02-13	04-03-13	6	D1	d51	29	10	10	9	0,34	0,34	0,66	0,31	release farm
2013	19-02-13	25-02-13	04-03-13	6	D2	d52	24	8	4	12	0,33	0,17	0,67	0,50	release farm
2013	19-02-13	25-02-13	04-03-13	6	D3	d53	15	6	2	7	0,40	0,13	0,60	0,47	release farm
2013	19-02-13	25-02-13	04-03-13	6	D4	d54	21	6	6	9	0,29	0,29	0,71	0,43	release farm
2013	19-02-13	25-02-13	04-03-13	6	D5	d55	17	4	5	8	0,24	0,29	0,76	0,47	release farm
2013	19-02-13	25-02-13	04-03-13	6	D6	d56	31	11	7	13	0,35	0,23	0,65	0,42	release farm
2013	19-02-13	25-02-13	04-03-13	6	D7	d57	22	6	6	10	0,27	0,27	0,73	0,45	release farm
2013	19-02-13	25-02-13	04-03-13	6	D8	d58	19	4	4	11	0,21	0,21	0,79	0,58	release farm
2013	19-02-13	25-02-13	04-03-13	6	D9	d59	20	8	7	6	0,40	0,35	0,65	0,30	release farm
2013	19-02-13	25-02-13	04-03-13	6	D10	d60	23	7	9	7	0,30	0,39	0,70	0,30	release farm
2013	04-03-13	09-03-13	16-03-13	7	D1	d61	19	4	3	12	0,21	0,16	0,79	0,63	release farm
2013	04-03-13	09-03-13	16-03-13	7	D2	d62	17	5	3	9	0,29	0,18	0,71	0,53	release farm
2013	04-03-13	09-03-13	16-03-13	7	D3	d63	14	4	3	7	0,29	0,21	0,71	0,50	release farm
2013	04-03-13	09-03-13	16-03-13	7	D4	d64	29	11	6	12	0,38	0,21	0,62	0,41	release farm
2013	04-03-13	09-03-13	16-03-13	7	D5	d65	19	8	3	8	0,42	0,16	0,58	0,42	release farm
2013	04-03-13	09-03-13	16-03-13	7	D6	d66	8	3	1	6	0,38	0,13	0,88	0,75	release farm
2013	04-03-13	09-03-13	16-03-13	7	D7	d67	28	8	5	15	0,29	0,18	0,71	0,54	release farm
2013	04-03-13	09-03-13	16-03-13	7	D8	d68	12	3	2	7	0,25	0,17	0,75	0,58	release farm
2013	04-03-13	09-03-13	16-03-13	7	D9	d69	9	0	3	6	0,00	0,33	1,00	0,67	release farm
2013	04-03-13	09-03-13	16-03-13	7	D10	d70	20	3	2	15	0,15	0,10	0,85	0,75	release farm
2013	16-03-13	21-03-13	28-03-13	8	D1	d71	24	7	4	13	0,29	0,17	0,71	0,54	release farm
2013	16-03-13	21-03-13	28-03-13	8	D2	d72	15	4	4	7	0,27	0,27	0,73	0,47	release farm
2013	16-03-13	21-03-13	28-03-13	8	D3	d73	19	5	3	11	0,26	0,16	0,74	0,58	release farm
2013	16-03-13	21-03-13	28-03-13	8	D4	d74	26	9	5	12	0,35	0,19	0,65	0,46	release farm
2013	16-03-13	21-03-13	28-03-13	8	D5	d75	12	4	3	5	0,33	0,25	0,67	0,42	release farm
2013	16-03-13	21-03-13	28-03-13	8	D6	d76	11	2	4	5	0,18	0,36	0,82	0,45	release farm
2013	16-03-13	21-03-13	28-03-13	8	D7	d77	9	0	1	8	0,00	0,11	1,00	0,89	release farm
2013	16-03-13	21-03-13	28-03-13	8	D8	d78	17	1	3	13	0,06	0,18	0,94	0,76	release farm

2013	16-03-13	21-03-13	28-03-13	8	D9	d79	12	0	2	10	0,00	0,17	1,00	0,83	release farm
2013	16-03-13	21-03-13	28-03-13	8	D10	d80	13	0	1	12	0,00	0,08	1,00	0,92	release farm
2013	28-03-13	04-04-13	11-04-13	9	D1	d81	10	1	2	7	0,10	0,20	0,90	0,70	release farm
2013	28-03-13	04-04-13	11-04-13	9	D2	d82	13	0	4	9	0,00	0,31	1,00	0,69	release farm
2013	28-03-13	04-04-13	11-04-13	9	D3	d83	10	1	1	8	0,10	0,10	0,90	0,80	release farm
2013	28-03-13	04-04-13	11-04-13	9	D4	d84	14	0	3	11	0,00	0,21	1,00	0,79	release farm
2013	28-03-13	04-04-13	11-04-13	9	D5	d85	13	2	4	7	0,15	0,31	0,85	0,54	release farm
2013	28-03-13	04-04-13	11-04-13	9	D6	d86	14	2	4	8	0,14	0,29	0,86	0,57	release farm
2013	28-03-13	04-04-13	11-04-13	9	D7	d87	10	2	2	6	0,20	0,20	0,80	0,60	release farm
2013	28-03-13	04-04-13	11-04-13	9	D8	d88	12	1	4	7	0,08	0,33	0,92	0,58	release farm
2013	28-03-13	04-04-13	11-04-13	9	D9	d89	10	1	2	7	0,10	0,20	0,90	0,70	release farm
2013	28-03-13	04-04-13	11-04-13	9	D10	d90	15	1	5	9	0,07	0,33	0,93	0,60	release farm
2013	11-04-13	16-04-13	23-04-13	10	D1	d91	17	6	3	8	0,35	0,18	0,65	0,47	release farm
2013	11-04-13	16-04-13	23-04-13	10	D2	d92	13	3	3	7	0,23	0,23	0,77	0,54	release farm
2013	11-04-13	16-04-13	23-04-13	10	D3	d93	9	1	1	7	0,11	0,11	0,89	0,78	release farm
2013	11-04-13	16-04-13	23-04-13	10	D4	d94	11	1	2	8	0,09	0,18	0,91	0,73	release farm
2013	11-04-13	16-04-13	23-04-13	10	D5	d95	8	0	1	7	0,00	0,13	1,00	0,88	release farm
2013	11-04-13	16-04-13	23-04-13	10	D6	d96	10	3	1	6	0,30	0,10	0,70	0,60	release farm
2013	11-04-13	16-04-13	23-04-13	10	D7	d97	6	0	1	5	0,00	0,17	1,00	0,83	release farm
2013	11-04-13	16-04-13	23-04-13	10	D8	d98	7	0	3	4	0,00	0,43	1,00	0,57	release farm
2013	11-04-13	16-04-13	23-04-13	10	D9	d99	5	0	1	4	0,00	0,20	1,00	0,80	release farm
2013	11-04-13	16-04-13	23-04-13	10	D10	d100	6	0	3	3	0,00	0,50	1,00	0,50	release farm
2013	23-04-13	28-04-13	05-05-13	11	D1	d101	10	2	2	6	0,20	0,20	0,80	0,60	release farm
2013	23-04-13	28-04-13	05-05-13	11	D2	d102	8	0	3	5	0,00	0,38	1,00	0,63	release farm
2013	23-04-13	28-04-13	05-05-13	11	D3	d103	9	1	3	5	0,11	0,33	0,89	0,56	release farm
2013	23-04-13	28-04-13	05-05-13	11	D4	d104	11	0	4	7	0,00	0,36	1,00	0,64	release farm
2013	23-04-13	28-04-13	05-05-13	11	D5	d105	7	0	0	7	0,00	0,00	1,00	1,00	release farm
2013	23-04-13	28-04-13	05-05-13	11	D6	d106	9	1	2	6	0,11	0,22	0,89	0,67	release farm
2013	23-04-13	28-04-13	05-05-13	11	D7	d107	10	0	4	6	0,00	0,40	1,00	0,60	release farm
2013	23-04-13	28-04-13	05-05-13	11	D8	d108	7	1	1	5	0,14	0,14	0,86	0,71	release farm
2013	23-04-13	28-04-13	05-05-13	11	D9	d109	8	1	1	6	0,13	0,13	0,88	0,75	release farm
2013	23-04-13	28-04-13	05-05-13	11	D10	d110	4	0	0	4	0,00	0,00	1,00	1,00	release farm
2013	05-05-13	10-05-13	17-05-13	12	D1	d111	6	1	1	4	0,17	0,17	0,83	0,67	release farm
2013	05-05-13	10-05-13	17-05-13	12	D2	d112	5	0	1	4	0,00	0,20	1,00	0,80	release farm
2013	05-05-13	10-05-13	17-05-13	12	D3	d113	10	2	1	7	0,20	0,10	0,80	0,70	release farm
2013	05-05-13	10-05-13	17-05-13	12	D4	d114	7	1	1	5	0,14	0,14	0,86	0,71	release farm
2013	05-05-13	10-05-13	17-05-13	12	D5	d115	6	1	2	3	0,17	0,33	0,83	0,50	release farm
2013	05-05-13	10-05-13	17-05-13	12	D6	d116	0	0	0	0	0,00	0,00	0,00	0,00	release farm
2013	05-05-13	10-05-13	17-05-13	12	D7	d117	7	1	1	5	0,14	0,14	0,86	0,71	release farm
2013	05-05-13	10-05-13	17-05-13	12	D8	d118	4	0	0	4	0,00	0,00	1,00	1,00	release farm

2013	05-05-13	10-05-13	17-05-13	12	D9	d119	3	0	1	2	0,00	0,33	1,00	0,67	release farm
2013	05-05-13	10-05-13	17-05-13	12	D10	d120	6	0	1	5	0,00	0,17	1,00	0,83	release farm
2013	17-05-13	23-05-13	30-05-13	13	D1	d121	5	1	1	3	0,20	0,20	0,80	0,60	release farm
2013	17-05-13	23-05-13	30-05-13	13	D2	d122	6	1	1	4	0,17	0,17	0,83	0,67	release farm
2013	17-05-13	23-05-13	30-05-13	13	D3	d123	4	0	1	3	0,00	0,25	1,00	0,75	release farm
2013	17-05-13	23-05-13	30-05-13	13	D4	d124	8	1	2	5	0,13	0,25	0,88	0,63	release farm
2013	17-05-13	23-05-13	30-05-13	13	D5	d125	4	0	1	3	0,00	0,25	1,00	0,75	release farm
2013	17-05-13	23-05-13	30-05-13	13	D6	d126	3	0	1	2	0,00	0,33	1,00	0,67	release farm
2013	17-05-13	23-05-13	30-05-13	13	D7	d127	0	0	0	0	0,00	0,00	0,00	0,00	release farm
2013	17-05-13	23-05-13	30-05-13	13	D8	d128	4	0	0	4	0,00	0,00	1,00	1,00	release farm
2013	17-05-13	23-05-13	30-05-13	13	D9	d129	0	0	0	0	0,00	0,00	0,00	0,00	release farm
2013	17-05-13	23-05-13	30-05-13	13	D10	d130	2	0	1	1	0,00	0,50	1,00	0,50	release farm
2014	20-12-13	26-12-13	02-01-14	1	D1	d131	22	10	3	9	0,45	0,14	0,55	0,41	release farm
2014	20-12-13	26-12-13	02-01-14	1	D2	d132	31	11	8	12	0,35	0,26	0,65	0,39	release farm
2014	20-12-13	26-12-13	02-01-14	1	D3	d133	21	9	3	9	0,43	0,14	0,57	0,43	release farm
2014	20-12-13	26-12-13	02-01-14	1	D4	d134	13	5	2	6	0,38	0,15	0,62	0,46	release farm
2014	20-12-13	26-12-13	02-01-14	1	D5	d135	16	7	2	7	0,44	0,13	0,56	0,44	release farm
2014	20-12-13	26-12-13	02-01-14	1	D6	d136	24	6	6	12	0,25	0,25	0,75	0,50	release farm
2014	20-12-13	26-12-13	02-01-14	1	D7	d137	16	5	3	8	0,31	0,19	0,69	0,50	release farm
2014	20-12-13	26-12-13	02-01-14	1	D8	d138	11	4	2	5	0,36	0,18	0,64	0,45	release farm
2014	20-12-13	26-12-13	02-01-14	1	D9	d139	19	11	2	6	0,58	0,11	0,42	0,32	release farm
2014	20-12-13	26-12-13	02-01-14	1	D10	d140	14	6	4	4	0,43	0,29	0,57	0,29	release farm
2014	02-01-14	07-01-14	14-01-14	2	D1	d141	23	6	10	7	0,26	0,43	0,74	0,30	release farm
2014	02-01-14	07-01-14	14-01-14	2	D2	d142	24	9	3	12	0,38	0,13	0,63	0,50	release farm
2014	02-01-14	07-01-14	14-01-14	2	D3	d143	19	6	4	9	0,32	0,21	0,68	0,47	release farm
2014	02-01-14	07-01-14	14-01-14	2	D4	d144	23	7	8	8	0,30	0,35	0,70	0,35	release farm
2014	02-01-14	07-01-14	14-01-14	2	D5	d145	16	4	7	5	0,25	0,44	0,75	0,31	release farm
2014	02-01-14	07-01-14	14-01-14	2	D6	d146	22	6	5	11	0,27	0,23	0,73	0,50	release farm
2014	02-01-14	07-01-14	14-01-14	2	D7	d147	16	7	3	6	0,44	0,19	0,56	0,38	release farm
2014	02-01-14	07-01-14	14-01-14	2	D8	d148	26	10	3	13	0,38	0,12	0,62	0,50	release farm
2014	02-01-14	07-01-14	14-01-14	2	D9	d149	24	14	3	7	0,58	0,13	0,42	0,29	release farm
2014	02-01-14	07-01-14	14-01-14	2	D10	d150	18	6	6	6	0,33	0,33	0,67	0,33	release farm
2014	14-01-14	19-01-14	26-01-14	3	D1	d151	23	7	7	9	0,30	0,30	0,70	0,39	release farm
2014	14-01-14	19-01-14	26-01-14	3	D2	d152	21	6	7	8	0,29	0,33	0,71	0,38	release farm
2014	14-01-14	19-01-14	26-01-14	3	D3	d153	15	4	3	8	0,27	0,20	0,73	0,53	release farm
2014	14-01-14	19-01-14	26-01-14	3	D4	d154	20	8	3	9	0,40	0,15	0,60	0,45	release farm
2014	14-01-14	19-01-14	26-01-14	3	D5	d155	19	5	6	8	0,26	0,32	0,74	0,42	release farm
2014	14-01-14	19-01-14	26-01-14	3	D6	d156	22	9	5	8	0,41	0,23	0,59	0,36	release farm
2014	14-01-14	19-01-14	26-01-14	3	D7	d157	20	7	4	9	0,35	0,20	0,65	0,45	release farm
2014	14-01-14	19-01-14	26-01-14	3	D8	d158	27	9	9	9	0,33	0,33	0,67	0,33	release farm

2014	14-01-14	19-01-14	26-01-14	3	D9	d159	17	6	4	7	0,35	0,24	0,65	0,41	release farm
2014	14-01-14	19-01-14	26-01-14	3	D10	d160	18	1	5	12	0,06	0,28	0,94	0,67	release farm
2014	26-01-14	31-01-14	07-02-14	4	D1	d161	16	5	5	6	0,31	0,31	0,69	0,38	release farm
2014	26-01-14	31-01-14	07-02-14	4	D2	d162	21	6	9	6	0,29	0,43	0,71	0,29	release farm
2014	26-01-14	31-01-14	07-02-14	4	D3	d163	12	4	2	6	0,33	0,17	0,67	0,50	release farm
2014	26-01-14	31-01-14	07-02-14	4	D4	d164	20	6	7	7	0,30	0,35	0,70	0,35	release farm
2014	26-01-14	31-01-14	07-02-14	4	D5	d165	14	5	4	5	0,36	0,29	0,64	0,36	release farm
2014	26-01-14	31-01-14	07-02-14	4	D6	d166	11	4	3	4	0,36	0,27	0,64	0,36	release farm
2014	26-01-14	31-01-14	07-02-14	4	D7	d167	15	6	3	6	0,40	0,20	0,60	0,40	release farm
2014	26-01-14	31-01-14	07-02-14	4	D8	d168	10	3	3	4	0,30	0,30	0,70	0,40	release farm
2014	26-01-14	31-01-14	07-02-14	4	D9	d169	13	2	5	6	0,15	0,38	0,85	0,46	release farm
2014	26-01-14	31-01-14	07-02-14	4	D10	d170	14	3	3	8	0,21	0,21	0,79	0,57	release farm
2014	26-01-14	31-01-14	07-02-14	5	D1	d171	22	7	7	8	0,32	0,32	0,68	0,36	release farm
2014	26-01-14	31-01-14	07-02-14	5	D2	d172	16	5	4	7	0,31	0,25	0,69	0,44	release farm
2014	26-01-14	31-01-14	07-02-14	5	D3	d173	13	6	4	3	0,46	0,31	0,54	0,23	release farm
2014	26-01-14	31-01-14	07-02-14	5	D4	d174	18	7	5	6	0,39	0,28	0,61	0,33	release farm
2014	26-01-14	31-01-14	07-02-14	5	D5	d175	10	2	4	4	0,20	0,40	0,80	0,40	release farm
2014	26-01-14	31-01-14	07-02-14	5	D6	d176	14	4	4	6	0,29	0,29	0,71	0,43	release farm
2014	26-01-14	31-01-14	07-02-14	5	D7	d177	15	6	2	7	0,40	0,13	0,60	0,47	release farm
2014	26-01-14	31-01-14	07-02-14	5	D8	d178	9	2	3	4	0,22	0,33	0,78	0,44	release farm
2014	26-01-14	31-01-14	07-02-14	5	D9	d179	10	3	2	5	0,30	0,20	0,70	0,50	release farm
2014	26-01-14	31-01-14	07-02-14	5	D10	d180	12	2	3	7	0,17	0,25	0,83	0,58	release farm
2014	19-02-14	25-02-14	04-03-14	6	D1	d181	14	4	5	5	0,29	0,36	0,71	0,36	release farm
2014	19-02-14	25-02-14	04-03-14	6	D2	d182	8	1	2	5	0,13	0,25	0,88	0,63	release farm
2014	19-02-14	25-02-14	04-03-14	6	D3	d183	17	5	5	7	0,29	0,29	0,71	0,41	release farm
2014	19-02-14	25-02-14	04-03-14	6	D4	d184	12	3	4	5	0,25	0,33	0,75	0,42	release farm
2014	19-02-14	25-02-14	04-03-14	6	D5	d185	18	5	5	8	0,28	0,28	0,72	0,44	release farm
2014	19-02-14	25-02-14	04-03-14	6	D6	d186	20	7	6	7	0,35	0,30	0,65	0,35	release farm
2014	19-02-14	25-02-14	04-03-14	6	D7	d187	14	6	2	6	0,43	0,14	0,57	0,43	release farm
2014	19-02-14	25-02-14	04-03-14	6	D8	d188	16	4	3	9	0,25	0,19	0,75	0,56	release farm
2014	19-02-14	25-02-14	04-03-14	6	D9	d189	10	2	3	5	0,20	0,30	0,80	0,50	release farm
2014	19-02-14	25-02-14	04-03-14	6	D10	d190	12	4	2	6	0,33	0,17	0,67	0,50	release farm
2014	04-03-14	09-03-14	16-03-14	7	D1	d191	12	2	5	5	0,17	0,42	0,83	0,42	release farm
2014	04-03-14	09-03-14	16-03-14	7	D2	d192	17	4	3	10	0,24	0,18	0,76	0,59	release farm
2014	04-03-14	09-03-14	16-03-14	7	D3	d193	10	2	4	4	0,20	0,40	0,80	0,40	release farm
2014	04-03-14	09-03-14	16-03-14	7	D4	d194	11	3	3	5	0,27	0,27	0,73	0,45	release farm
2014	04-03-14	09-03-14	16-03-14	7	D5	d195	13	2	5	6	0,15	0,38	0,85	0,46	release farm
2014	04-03-14	09-03-14	16-03-14	7	D6	d196	10	3	3	4	0,30	0,30	0,70	0,40	release farm
2014	04-03-14	09-03-14	16-03-14	7	D7	d197	12	2	4	6	0,17	0,33	0,83	0,50	release farm
2014	04-03-14	09-03-14	16-03-14	7	D8	d198	10	1	3	6	0,10	0,30	0,90	0,60	release farm

2014	04-03-14	09-03-14	16-03-14	7	D9	d199	11	2	2	7	0,18	0,18	0,82	0,64	release farm
2014	04-03-14	09-03-14	16-03-14	7	D10	d200	10	1	3	6	0,10	0,30	0,90	0,60	release farm
2014	16-03-14	21-03-14	28-03-14	8	D1	d201	13	1	4	8	0,08	0,31	0,92	0,62	release farm
2014	16-03-14	21-03-14	28-03-14	8	D2	d202	9	2	5	5	0,22	0,56	1,11	0,56	release farm
2014	16-03-14	21-03-14	28-03-14	8	D3	d203	21	7	6	8	0,33	0,29	0,67	0,38	release farm
2014	16-03-14	21-03-14	28-03-14	8	D4	d204	14	6	4	4	0,43	0,29	0,57	0,29	release farm
2014	16-03-14	21-03-14	28-03-14	8	D5	d205	10	1	4	5	0,10	0,40	0,90	0,50	release farm
2014	16-03-14	21-03-14	28-03-14	8	D6	d206	16	3	6	7	0,19	0,38	0,81	0,44	release farm
2014	16-03-14	21-03-14	28-03-14	8	D7	d207	11	2	4	5	0,18	0,36	0,82	0,45	release farm
2014	16-03-14	21-03-14	28-03-14	8	D8	d208	10	2	4	4	0,20	0,40	0,80	0,40	release farm
2014	16-03-14	21-03-14	28-03-14	8	D9	d209	12	3	3	6	0,25	0,25	0,75	0,50	release farm
2014	16-03-14	21-03-14	28-03-14	8	D10	d210	14	2	1	11	0,14	0,07	0,86	0,79	release farm
2014	28-03-14	04-04-14	11-04-14	9	D1	d211	18	5	6	7	0,28	0,33	0,72	0,39	release farm
2014	28-03-14	04-04-14	11-04-14	9	D2	d212	14	4	5	5	0,29	0,36	0,71	0,36	release farm
2014	28-03-14	04-04-14	11-04-14	9	D3	d213	9	2	3	4	0,22	0,33	0,78	0,44	release farm
2014	28-03-14	04-04-14	11-04-14	9	D4	d214	11	2	5	4	0,18	0,45	0,82	0,36	release farm
2014	28-03-14	04-04-14	11-04-14	9	D5	d215	12	3	3	6	0,25	0,25	0,75	0,50	release farm
2014	28-03-14	04-04-14	11-04-14	9	D6	d216	10	1	5	4	0,10	0,50	0,90	0,40	release farm
2014	28-03-14	04-04-14	11-04-14	9	D7	d217	9	1	4	4	0,11	0,44	0,89	0,44	release farm
2014	28-03-14	04-04-14	11-04-14	9	D8	d218	10	1	3	6	0,10	0,30	0,90	0,60	release farm
2014	28-03-14	04-04-14	11-04-14	9	D9	d219	11	1	3	7	0,09	0,27	0,91	0,64	release farm
2014	28-03-14	04-04-14	11-04-14	9	D10	d220	10	1	3	6	0,10	0,30	0,90	0,60	release farm
2014	11-04-14	16-04-14	23-04-14	10	D1	d221	14	2	4	8	0,14	0,29	0,86	0,57	release farm
2014	11-04-14	16-04-14	23-04-14	10	D2	d222	12	3	4	5	0,25	0,33	0,75	0,42	release farm
2014	11-04-14	16-04-14	23-04-14	10	D3	d223	11	0	5	6	0,00	0,45	1,00	0,55	release farm
2014	11-04-14	16-04-14	23-04-14	10	D4	d224	9	0	4	5	0,00	0,44	1,00	0,56	release farm
2014	11-04-14	16-04-14	23-04-14	10	D5	d225	10	1	2	7	0,10	0,20	0,90	0,70	release farm
2014	11-04-14	16-04-14	23-04-14	10	D6	d226	8	0	3	5	0,00	0,38	1,00	0,63	release farm
2014	11-04-14	16-04-14	23-04-14	10	D7	d227	11	0	3	8	0,00	0,27	1,00	0,73	release farm
2014	11-04-14	16-04-14	23-04-14	10	D8	d228	9	0	3	6	0,00	0,33	1,00	0,67	release farm
2014	11-04-14	16-04-14	23-04-14	10	D9	d229	10	0	2	8	0,00	0,20	1,00	0,80	release farm
2014	11-04-14	16-04-14	23-04-14	10	D10	d230	11	1	2	8	0,09	0,18	0,91	0,73	release farm
2014	23-04-14	28-04-14	05-05-14	11	D1	d231	6	0	3	3	0,00	0,50	1,00	0,50	release farm
2014	23-04-14	28-04-14	05-05-14	11	D2	d232	8	1	2	5	0,13	0,25	0,88	0,63	release farm
2014	23-04-14	28-04-14	05-05-14	11	D3	d233	9	0	3	6	0,00	0,33	1,00	0,67	release farm
2014	23-04-14	28-04-14	05-05-14	11	D4	d234	6	1	2	3	0,17	0,33	0,83	0,50	release farm
2014	23-04-14	28-04-14	05-05-14	11	D5	d235	12	1	4	7	0,08	0,33	0,92	0,58	release farm
2014	23-04-14	28-04-14	05-05-14	11	D6	d236	9	1	1	7	0,11	0,11	0,89	0,78	release farm
2014	23-04-14	28-04-14	05-05-14	11	D7	d237	8	0	3	5	0,00	0,38	1,00	0,63	release farm
2014	23-04-14	28-04-14	05-05-14	11	D8	d238	6	0	1	5	0,00	0,17	1,00	0,83	release farm

2014	23-04-14	28-04-14	05-05-14	11	D9	d239	5	0	1	4	0,00	0,20	1,00	0,80	release farm
2014	23-04-14	28-04-14	05-05-14	11	D10	d240	0	0	0	0	0,00	0,00	0,00	0,00	release farm
2014	05-05-14	10-05-14	17-05-14	12	D1	d241	10	1	4	5	0,10	0,40	0,90	0,50	release farm
2014	05-05-14	10-05-14	17-05-14	12	D2	d242	8	0	2	6	0,00	0,25	1,00	0,75	release farm
2014	05-05-14	10-05-14	17-05-14	12	D3	d243	6	0	1	5	0,00	0,17	1,00	0,83	release farm
2014	05-05-14	10-05-14	17-05-14	12	D4	d244	8	0	2	6	0,00	0,25	1,00	0,75	release farm
2014	05-05-14	10-05-14	17-05-14	12	D5	d245	6	1	2	3	0,17	0,33	0,83	0,50	release farm
2014	05-05-14	10-05-14	17-05-14	12	D6	d246	8	0	2	6	0,00	0,25	1,00	0,75	release farm
2014	05-05-14	10-05-14	17-05-14	12	D7	d247	0	0	0	0	0,00	0,00	0,00	0,00	release farm
2014	05-05-14	10-05-14	17-05-14	12	D8	d248	8	0	3	5	0,00	0,38	1,00	0,63	release farm
2014	05-05-14	10-05-14	17-05-14	12	D9	d249	6	0	2	4	0,00	0,33	1,00	0,67	release farm
2014	05-05-14	10-05-14	17-05-14	12	D10	d250	0	0	0	0	0,00	0,00	0,00	0,00	release farm
2014	17-05-14	23-05-14	30-05-14	13	D1	d251	8	1	2	5	0,13	0,25	0,88	0,63	release farm
2014	17-05-14	23-05-14	30-05-14	13	D2	d252	9	0	3	6	0,00	0,33	1,00	0,67	release farm
2014	17-05-14	23-05-14	30-05-14	13	D3	d253	6	0	2	4	0,00	0,33	1,00	0,67	release farm
2014	17-05-14	23-05-14	30-05-14	13	D4	d254	4	0	1	3	0,00	0,25	1,00	0,75	release farm
2014	17-05-14	23-05-14	30-05-14	13	D5	d255	6	0	2	4	0,00	0,33	1,00	0,67	release farm
2014	17-05-14	23-05-14	30-05-14	13	D6	d256	4	0	1	3	0,00	0,25	1,00	0,75	release farm
2014	17-05-14	23-05-14	30-05-14	13	D7	d257	9	0	2	7	0,00	0,22	1,00	0,78	release farm
2014	17-05-14	23-05-14	30-05-14	13	D8	d258	0	0	0	0	0,00	0,00	0,00	0,00	release farm
2014	17-05-14	23-05-14	30-05-14	13	D9	d259	0	0	0	0	0,00	0,00	0,00	0,00	release farm
2014	17-05-14	23-05-14	30-05-14	13	D10	d260	0	0	0	0	0,00	0,00	0,00	0,00	release farm
2013	21-12-12	26-12-12	02-01-13	1	D1	d261	28	15	0	13	0,54	0,00	0,46	0,46	non release farm
2013	21-12-12	26-12-12	02-01-13	1	D2	d262	11	7	0	4	0,64	0,00	0,36	0,36	non release farm
2013	21-12-12	26-12-12	02-01-13	1	D3	d263	20	12	0	8	0,60	0,00	0,40	0,40	non release farm
2013	21-12-12	26-12-12	02-01-13	1	D4	d264	16	10	0	6	0,63	0,00	0,38	0,38	non release farm
2013	21-12-12	26-12-12	02-01-13	1	D5	d265	23	18	0	5	0,78	0,00	0,22	0,22	non release farm
2013	21-12-12	26-12-12	02-01-13	1	D6	d266	22	15	0	7	0,68	0,00	0,32	0,32	non release farm
2013	21-12-12	26-12-12	02-01-13	1	D7	d267	19	11	0	8	0,58	0,00	0,42	0,42	non release farm
2013	21-12-12	26-12-12	02-01-13	1	D8	d268	24	14	0	10	0,58	0,00	0,42	0,42	non release farm
2013	21-12-12	26-12-12	02-01-13	1	D9	d269	21	12	0	9	0,57	0,00	0,43	0,43	non release farm
2013	21-12-12	26-12-12	02-01-13	1	D10	d270	17	10	0	7	0,59	0,00	0,41	0,41	non release farm
2013	02-01-13	07-01-13	14-01-13	2	D1	d271	32	18	0	14	0,56	0,00	0,44	0,44	non release farm
2013	02-01-13	07-01-13	14-01-13	2	D2	d272	21	11	0	10	0,52	0,00	0,48	0,48	non release farm
2013	02-01-13	07-01-13	14-01-13	2	D3	d273	33	20	0	13	0,61	0,00	0,39	0,39	non release farm
2013	02-01-13	07-01-13	14-01-13	2	D4	d274	19	12	0	7	0,63	0,00	0,37	0,37	non release farm
2013	02-01-13	07-01-13	14-01-13	2	D5	d275	35	18	0	17	0,51	0,00	0,49	0,49	non release farm
2013	02-01-13	07-01-13	14-01-13	2	D6	d276	11	6	0	5	0,55	0,00	0,45	0,45	non release farm
2013	02-01-13	07-01-13	14-01-13	2	D7	d277	17	10	0	7	0,59	0,00	0,41	0,41	non release farm
2013	02-01-13	07-01-13	14-01-13	2	D8	d278	29	16	0	13	0,55	0,00	0,45	0,45	non release farm

2013	02-01-13	07-01-13	14-01-13	2	D9	d279	18	11	0	7	0,61	0,00	0,39	0,39	non release farm
2013	02-01-13	07-01-13	14-01-13	2	D10	d280	22	12	0	10	0,55	0,00	0,45	0,45	non release farm
2013	14-01-13	19-01-13	26-01-13	3	D1	d281	30	17	0	13	0,57	0,00	0,43	0,43	non release farm
2013	14-01-13	19-01-13	26-01-13	3	D2	d282	26	19	0	7	0,73	0,00	0,27	0,27	non release farm
2013	14-01-13	19-01-13	26-01-13	3	D3	d283	14	8	0	6	0,57	0,00	0,43	0,43	non release farm
2013	14-01-13	19-01-13	26-01-13	3	D4	d284	28	15	0	13	0,54	0,00	0,46	0,46	non release farm
2013	14-01-13	19-01-13	26-01-13	3	D5	d285	10	6	0	4	0,60	0,00	0,40	0,40	non release farm
2013	14-01-13	19-01-13	26-01-13	3	D6	d286	9	4	0	5	0,44	0,00	0,56	0,56	non release farm
2013	14-01-13	19-01-13	26-01-13	3	D7	d287	13	7	0	6	0,54	0,00	0,46	0,46	non release farm
2013	14-01-13	19-01-13	26-01-13	3	D8	d288	18	10	0	8	0,56	0,00	0,44	0,44	non release farm
2013	14-01-13	19-01-13	26-01-13	3	D9	d289	13	7	0	6	0,54	0,00	0,46	0,46	non release farm
2013	14-01-13	19-01-13	26-01-13	3	D10	d290	21	12	0	9	0,57	0,00	0,43	0,43	non release farm
2013	26-01-13	31-01-13	07-02-13	4	D1	d291	26	19	0	7	0,73	0,00	0,27	0,27	non release farm
2013	26-01-13	31-01-13	07-02-13	4	D2	d292	19	10	0	9	0,53	0,00	0,47	0,47	non release farm
2013	26-01-13	31-01-13	07-02-13	4	D3	d293	30	19	0	11	0,63	0,00	0,37	0,37	non release farm
2013	26-01-13	31-01-13	07-02-13	4	D4	d294	13	8	0	5	0,62	0,00	0,38	0,38	non release farm
2013	26-01-13	31-01-13	07-02-13	4	D5	d295	17	14	0	3	0,82	0,00	0,18	0,18	non release farm
2013	26-01-13	31-01-13	07-02-13	4	D6	d296	15	11	0	4	0,73	0,00	0,27	0,27	non release farm
2013	26-01-13	31-01-13	07-02-13	4	D7	d297	18	13	0	5	0,72	0,00	0,28	0,28	non release farm
2013	26-01-13	31-01-13	07-02-13	4	D8	d298	22	12	0	10	0,55	0,00	0,45	0,45	non release farm
2013	26-01-13	31-01-13	07-02-13	4	D9	d299	19	11	0	8	0,58	0,00	0,42	0,42	non release farm
2013	26-01-13	31-01-13	07-02-13	4	D10	d300	24	11	0	13	0,46	0,00	0,54	0,54	non release farm
2013	07-02-13	12-02-13	19-02-13	5	D1	d301	22	13	0	9	0,59	0,00	0,41	0,41	non release farm
2013	07-02-13	12-02-13	19-02-13	5	D2	d302	30	18	0	12	0,60	0,00	0,40	0,40	non release farm
2013	07-02-13	12-02-13	19-02-13	5	D3	d303	27	16	0	11	0,59	0,00	0,41	0,41	non release farm
2013	07-02-13	12-02-13	19-02-13	5	D4	d304	24	14	0	10	0,58	0,00	0,42	0,42	non release farm
2013	07-02-13	12-02-13	19-02-13	5	D5	d305	21	13	0	8	0,62	0,00	0,38	0,38	non release farm
2013	07-02-13	12-02-13	19-02-13	5	D6	d306	25	15	0	10	0,60	0,00	0,40	0,40	non release farm
2013	07-02-13	12-02-13	19-02-13	5	D7	d307	32	17	0	15	0,53	0,00	0,47	0,47	non release farm
2013	07-02-13	12-02-13	19-02-13	5	D8	d308	20	12	0	8	0,60	0,00	0,40	0,40	non release farm
2013	07-02-13	12-02-13	19-02-13	5	D9	d309	24	15	0	9	0,63	0,00	0,38	0,38	non release farm
2013	07-02-13	12-02-13	19-02-13	5	D10	d310	16	11	0	5	0,69	0,00	0,31	0,31	non release farm
2013	19-02-13	25-02-13	04-03-13	6	D1	d311	26	15	0	11	0,58	0,00	0,42	0,42	non release farm
2013	19-02-13	25-02-13	04-03-13	6	D2	d312	30	16	0	14	0,53	0,00	0,47	0,47	non release farm
2013	19-02-13	25-02-13	04-03-13	6	D3	d313	27	15	0	12	0,56	0,00	0,44	0,44	non release farm
2013	19-02-13	25-02-13	04-03-13	6	D4	d314	19	12	0	7	0,63	0,00	0,37	0,37	non release farm
2013	19-02-13	25-02-13	04-03-13	6	D5	d315	21	15	0	6	0,71	0,00	0,29	0,29	non release farm
2013	19-02-13	25-02-13	04-03-13	6	D6	d316	28	19	0	9	0,68	0,00	0,32	0,32	non release farm
2013	19-02-13	25-02-13	04-03-13	6	D7	d317	25	13	0	12	0,52	0,00	0,48	0,48	non release farm
2013	19-02-13	25-02-13	04-03-13	6	D8	d318	37	23	0	14	0,62	0,00	0,38	0,38	non release farm

2013	19-02-13	25-02-13	04-03-13	6	D9	d319	28	10	0	8	0,36	0,00	0,29	0,29	non release farm
2013	19-02-13	25-02-13	04-03-13	6	D10	d320	26	15	0	11	0,58	0,00	0,42	0,42	non release farm
2013	04-03-13	09-03-13	16-03-13	7	D1	d321	37	20	0	17	0,54	0,00	0,46	0,46	non release farm
2013	04-03-13	09-03-13	16-03-13	7	D2	d322	32	20	0	12	0,63	0,00	0,38	0,38	non release farm
2013	04-03-13	09-03-13	16-03-13	7	D3	d323	21	14	0	7	0,67	0,00	0,33	0,33	non release farm
2013	04-03-13	09-03-13	16-03-13	7	D4	d324	31	21	0	10	0,68	0,00	0,32	0,32	non release farm
2013	04-03-13	09-03-13	16-03-13	7	D5	d325	25	15	0	10	0,60	0,00	0,40	0,40	non release farm
2013	04-03-13	09-03-13	16-03-13	7	D6	d326	29	18	0	11	0,62	0,00	0,38	0,38	non release farm
2013	04-03-13	09-03-13	16-03-13	7	D7	d327	28	16	0	12	0,57	0,00	0,43	0,43	non release farm
2013	04-03-13	09-03-13	16-03-13	7	D8	d328	41	25	0	16	0,61	0,00	0,39	0,39	non release farm
2013	04-03-13	09-03-13	16-03-13	7	D9	d329	35	23	0	12	0,66	0,00	0,34	0,34	non release farm
2013	04-03-13	09-03-13	16-03-13	7	D10	d330	26	19	0	7	0,73	0,00	0,27	0,27	non release farm
2013	16-03-13	21-03-13	28-03-13	8	D1	d331	22	15	0	7	0,68	0,00	0,32	0,32	non release farm
2013	16-03-13	21-03-13	28-03-13	8	D2	d332	48	30	0	18	0,63	0,00	0,38	0,38	non release farm
2013	16-03-13	21-03-13	28-03-13	8	D3	d333	23	13	0	10	0,57	0,00	0,43	0,43	non release farm
2013	16-03-13	21-03-13	28-03-13	8	D4	d334	34	24	0	10	0,71	0,00	0,29	0,29	non release farm
2013	16-03-13	21-03-13	28-03-13	8	D5	d335	24	13	0	11	0,54	0,00	0,46	0,46	non release farm
2013	16-03-13	21-03-13	28-03-13	8	D6	d336	25	13	0	12	0,52	0,00	0,48	0,48	non release farm
2013	16-03-13	21-03-13	28-03-13	8	D7	d337	16	7	0	9	0,44	0,00	0,56	0,56	non release farm
2013	16-03-13	21-03-13	28-03-13	8	D8	d338	29	21	0	8	0,72	0,00	0,28	0,28	non release farm
2013	16-03-13	21-03-13	28-03-13	8	D9	d339	26	19	0	7	0,73	0,00	0,27	0,27	non release farm
2013	16-03-13	21-03-13	28-03-13	8	D10	d340	21	13	0	8	0,62	0,00	0,38	0,38	non release farm
2013	28-03-13	04-04-13	11-04-13	9	D1	d341	28	16	0	12	0,57	0,00	0,43	0,43	non release farm
2013	28-03-13	04-04-13	11-04-13	9	D2	d342	17	8	0	9	0,47	0,00	0,53	0,53	non release farm
2013	28-03-13	04-04-13	11-04-13	9	D3	d343	24	12	0	12	0,50	0,00	0,50	0,50	non release farm
2013	28-03-13	04-04-13	11-04-13	9	D4	d344	27	14	0	13	0,52	0,00	0,48	0,48	non release farm
2013	28-03-13	04-04-13	11-04-13	9	D5	d345	35	21	0	14	0,60	0,00	0,40	0,40	non release farm
2013	28-03-13	04-04-13	11-04-13	9	D6	d346	13	7	0	6	0,54	0,00	0,46	0,46	non release farm
2013	28-03-13	04-04-13	11-04-13	9	D7	d347	21	11	0	10	0,52	0,00	0,48	0,48	non release farm
2013	28-03-13	04-04-13	11-04-13	9	D8	d348	32	18	0	14	0,56	0,00	0,44	0,44	non release farm
2013	28-03-13	04-04-13	11-04-13	9	D9	d349	23	16	0	7	0,70	0,00	0,30	0,30	non release farm
2013	28-03-13	04-04-13	11-04-13	9	D10	d350	19	13	0	6	0,68	0,00	0,32	0,32	non release farm
2013	11-04-13	16-04-13	23-04-13	10	D1	d351	20	12	0	8	0,60	0,00	0,40	0,40	non release farm
2013	11-04-13	16-04-13	23-04-13	10	D2	d352	14	8	0	6	0,57	0,00	0,43	0,43	non release farm
2013	11-04-13	16-04-13	23-04-13	10	D3	d353	15	7	0	8	0,47	0,00	0,53	0,53	non release farm
2013	11-04-13	16-04-13	23-04-13	10	D4	d354	23	12	0	11	0,52	0,00	0,48	0,48	non release farm
2013	11-04-13	16-04-13	23-04-13	10	D5	d355	20	11	0	9	0,55	0,00	0,45	0,45	non release farm
2013	11-04-13	16-04-13	23-04-13	10	D6	d356	17	9	0	8	0,53	0,00	0,47	0,47	non release farm
2013	11-04-13	16-04-13	23-04-13	10	D7	d357	13	8	0	5	0,62	0,00	0,38	0,38	non release farm
2013	11-04-13	16-04-13	23-04-13	10	D8	d358	11	5	0	6	0,45	0,00	0,55	0,55	non release farm

2013	11-04-13	16-04-13	23-04-13	10	D9	d359	13	6	0	7	0,46	0,00	0,54	0,54	non release farm
2013	11-04-13	16-04-13	23-04-13	10	D10	d360	10	4	0	6	0,40	0,00	0,60	0,60	non release farm
2013	23-04-13	28-04-13	05-05-13	11	D1	d361	13	7	0	6	0,54	0,00	0,46	0,46	non release farm
2013	23-04-13	28-04-13	05-05-13	11	D2	d362	9	5	0	4	0,56	0,00	0,44	0,44	non release farm
2013	23-04-13	28-04-13	05-05-13	11	D3	d363	7	4	0	3	0,57	0,00	0,43	0,43	non release farm
2013	23-04-13	28-04-13	05-05-13	11	D4	d364	12	5	0	7	0,42	0,00	0,58	0,58	non release farm
2013	23-04-13	28-04-13	05-05-13	11	D5	d365	16	4	0	12	0,25	0,00	0,75	0,75	non release farm
2013	23-04-13	28-04-13	05-05-13	11	D6	d366	8	3	0	5	0,38	0,00	0,63	0,63	non release farm
2013	23-04-13	28-04-13	05-05-13	11	D7	d367	13	8	0	5	0,62	0,00	0,38	0,38	non release farm
2013	23-04-13	28-04-13	05-05-13	11	D8	d368	14	7	0	7	0,50	0,00	0,50	0,50	non release farm
2013	23-04-13	28-04-13	05-05-13	11	D9	d369	18	14	0	4	0,78	0,00	0,22	0,22	non release farm
2013	23-04-13	28-04-13	05-05-13	11	D10	d370	8	3	0	5	0,38	0,00	0,63	0,63	non release farm
2013	05-05-13	10-05-13	17-05-13	12	D1	d371	10	6	0	4	0,60	0,00	0,40	0,40	non release farm
2013	05-05-13	10-05-13	17-05-13	12	D2	d372	12	7	0	5	0,58	0,00	0,42	0,42	non release farm
2013	05-05-13	10-05-13	17-05-13	12	D3	d373	17	12	0	5	0,71	0,00	0,29	0,29	non release farm
2013	05-05-13	10-05-13	17-05-13	12	D4	d374	14	10	0	4	0,71	0,00	0,29	0,29	non release farm
2013	05-05-13	10-05-13	17-05-13	12	D5	d375	11	7	0	4	0,64	0,00	0,36	0,36	non release farm
2013	05-05-13	10-05-13	17-05-13	12	D6	d376	9	3	0	6	0,33	0,00	0,67	0,67	non release farm
2013	05-05-13	10-05-13	17-05-13	12	D7	d377	15	4	0	11	0,27	0,00	0,73	0,73	non release farm
2013	05-05-13	10-05-13	17-05-13	12	D8	d378	12	3	0	9	0,25	0,00	0,75	0,75	non release farm
2013	05-05-13	10-05-13	17-05-13	12	D9	d379	19	7	0	12	0,37	0,00	0,63	0,63	non release farm
2013	05-05-13	10-05-13	17-05-13	12	D10	d380	21	8	0	13	0,38	0,00	0,62	0,62	non release farm
2013	17-05-13	23-05-13	30-05-13	13	D1	d381	14	9	0	5	0,64	0,00	0,36	0,36	non release farm
2013	17-05-13	23-05-13	30-05-13	13	D2	d382	9	5	0	4	0,56	0,00	0,44	0,44	non release farm
2013	17-05-13	23-05-13	30-05-13	13	D3	d383	12	9	0	3	0,75	0,00	0,25	0,25	non release farm
2013	17-05-13	23-05-13	30-05-13	13	D4	d384	8	6	0	2	0,75	0,00	0,25	0,25	non release farm
2013	17-05-13	23-05-13	30-05-13	13	D5	d385	26	17	0	9	0,65	0,00	0,35	0,35	non release farm
2013	17-05-13	23-05-13	30-05-13	13	D6	d386	11	5	0	6	0,45	0,00	0,55	0,55	non release farm
2013	17-05-13	23-05-13	30-05-13	13	D7	d387	10	1	0	9	0,10	0,00	0,90	0,90	non release farm
2013	17-05-13	23-05-13	30-05-13	13	D8	d388	13	8	0	5	0,62	0,00	0,38	0,38	non release farm
2013	17-05-13	23-05-13	30-05-13	13	D9	d389	18	4	0	14	0,22	0,00	0,78	0,78	non release farm
2013	17-05-13	23-05-13	30-05-13	13	D10	d390	10	2	0	8	0,20	0,00	0,80	0,80	non release farm
2014	20-12-13	26-12-13	02-01-14	1	D1	d391	34	23	0	11	0,68	0,00	0,32	0,32	non release farm
2014	20-12-13	26-12-13	02-01-14	1	D2	d392	21	11	0	10	0,52	0,00	0,48	0,48	non release farm
2014	20-12-13	26-12-13	02-01-14	1	D3	d393	31	21	0	10	0,68	0,00	0,32	0,32	non release farm
2014	20-12-13	26-12-13	02-01-14	1	D4	d394	22	13	0	9	0,59	0,00	0,41	0,41	non release farm
2014	20-12-13	26-12-13	02-01-14	1	D5	d395	24	14	0	10	0,58	0,00	0,42	0,42	non release farm
2014	20-12-13	26-12-13	02-01-14	1	D6	d396	23	9	0	14	0,39	0,00	0,61	0,61	non release farm
2014	20-12-13	26-12-13	02-01-14	1	D7	d397	30	15	0	15	0,50	0,00	0,50	0,50	non release farm
2014	20-12-13	26-12-13	02-01-14	1	D8	d398	20	11	0	9	0,55	0,00	0,45	0,45	non release farm

2014	20-12-13	26-12-13	02-01-14	1	D9	d399	32	19	0	13	0,59	0,00	0,41	0,41	non release farm
2014	20-12-13	26-12-13	02-01-14	1	D10	d400	26	12	0	14	0,46	0,00	0,54	0,54	non release farm
2014	02-01-14	07-01-14	14-01-14	2	D1	d401	24	13	0	11	0,54	0,00	0,46	0,46	non release farm
2014	02-01-14	07-01-14	14-01-14	2	D2	d402	48	29	0	19	0,60	0,00	0,40	0,40	non release farm
2014	02-01-14	07-01-14	14-01-14	2	D3	d403	21	13	0	8	0,62	0,00	0,38	0,38	non release farm
2014	02-01-14	07-01-14	14-01-14	2	D4	d404	30	18	0	12	0,60	0,00	0,40	0,40	non release farm
2014	02-01-14	07-01-14	14-01-14	2	D5	d405	22	13	0	9	0,59	0,00	0,41	0,41	non release farm
2014	02-01-14	07-01-14	14-01-14	2	D6	d406	20	14	0	6	0,70	0,00	0,30	0,30	non release farm
2014	02-01-14	07-01-14	14-01-14	2	D7	d407	25	15	0	10	0,60	0,00	0,40	0,40	non release farm
2014	02-01-14	07-01-14	14-01-14	2	D8	d408	14	9	0	5	0,64	0,00	0,36	0,36	non release farm
2014	02-01-14	07-01-14	14-01-14	2	D9	d409	22	15	0	7	0,68	0,00	0,32	0,32	non release farm
2014	02-01-14	07-01-14	14-01-14	2	D10	d410	16	10	0	6	0,63	0,00	0,38	0,38	non release farm
2014	14-01-14	19-01-14	26-01-14	3	D1	d411	26	16	0	10	0,62	0,00	0,38	0,38	non release farm
2014	14-01-14	19-01-14	26-01-14	3	D2	d412	18	10	0	8	0,56	0,00	0,44	0,44	non release farm
2014	14-01-14	19-01-14	26-01-14	3	D3	d413	41	27	0	14	0,66	0,00	0,34	0,34	non release farm
2014	14-01-14	19-01-14	26-01-14	3	D4	d414	24	16	0	8	0,67	0,00	0,33	0,33	non release farm
2014	14-01-14	19-01-14	26-01-14	3	D5	d415	32	19	0	13	0,59	0,00	0,41	0,41	non release farm
2014	14-01-14	19-01-14	26-01-14	3	D6	d416	19	12	0	7	0,63	0,00	0,37	0,37	non release farm
2014	14-01-14	19-01-14	26-01-14	3	D7	d417	24	11	0	13	0,46	0,00	0,54	0,54	non release farm
2014	14-01-14	19-01-14	26-01-14	3	D8	d418	20	13	0	7	0,65	0,00	0,35	0,35	non release farm
2014	14-01-14	19-01-14	26-01-14	3	D9	d419	18	10	0	8	0,56	0,00	0,44	0,44	non release farm
2014	14-01-14	19-01-14	26-01-14	3	D10	d420	28	17	0	11	0,61	0,00	0,39	0,39	non release farm
2014	26-01-14	31-01-14	07-02-14	4	D1	d421	32	20	0	12	0,63	0,00	0,38	0,38	non release farm
2014	26-01-14	31-01-14	07-02-14	4	D2	d422	41	26	0	15	0,63	0,00	0,37	0,37	non release farm
2014	26-01-14	31-01-14	07-02-14	4	D3	d423	25	17	0	8	0,68	0,00	0,32	0,32	non release farm
2014	26-01-14	31-01-14	07-02-14	4	D4	d424	32	17	0	15	0,53	0,00	0,47	0,47	non release farm
2014	26-01-14	31-01-14	07-02-14	4	D5	d425	22	10	0	12	0,45	0,00	0,55	0,55	non release farm
2014	26-01-14	31-01-14	07-02-14	4	D6	d426	27	16	0	11	0,59	0,00	0,41	0,41	non release farm
2014	26-01-14	31-01-14	07-02-14	4	D7	d427	22	13	0	9	0,59	0,00	0,41	0,41	non release farm
2014	26-01-14	31-01-14	07-02-14	4	D8	d428	16	9	0	7	0,56	0,00	0,44	0,44	non release farm
2014	26-01-14	31-01-14	07-02-14	4	D9	d429	24	15	0	9	0,63	0,00	0,38	0,38	non release farm
2014	26-01-14	31-01-14	07-02-14	4	D10	d430	30	13	0	17	0,43	0,00	0,57	0,57	non release farm
2014	26-01-14	31-01-14	07-02-14	5	D1	d431	12	7	0	5	0,58	0,00	0,42	0,42	non release farm
2014	26-01-14	31-01-14	07-02-14	5	D2	d432	21	12	0	9	0,57	0,00	0,43	0,43	non release farm
2014	26-01-14	31-01-14	07-02-14	5	D3	d433	14	6	0	8	0,43	0,00	0,57	0,57	non release farm
2014	26-01-14	31-01-14	07-02-14	5	D4	d434	32	18	0	14	0,56	0,00	0,44	0,44	non release farm
2014	26-01-14	31-01-14	07-02-14	5	D5	d435	26	15	0	11	0,58	0,00	0,42	0,42	non release farm
2014	26-01-14	31-01-14	07-02-14	5	D6	d436	17	9	0	8	0,53	0,00	0,47	0,47	non release farm
2014	26-01-14	31-01-14	07-02-14	5	D7	d437	27	14	0	10	0,52	0,00	0,37	0,37	non release farm
2014	26-01-14	31-01-14	07-02-14	5	D8	d438	16	8	0	8	0,50	0,00	0,50	0,50	non release farm

2014	26-01-14	31-01-14	07-02-14	5	D9	d439	24	14	0	10	0,58	0,00	0,42	0,42	non release farm
2014	26-01-14	31-01-14	07-02-14	5	D10	d440	21	12	0	9	0,57	0,00	0,43	0,43	non release farm
2014	19-02-14	25-02-14	04-03-14	6	D1	d441	14	8	0	6	0,57	0,00	0,43	0,43	non release farm
2014	19-02-14	25-02-14	04-03-14	6	D2	d442	22	14	0	8	0,64	0,00	0,36	0,36	non release farm
2014	19-02-14	25-02-14	04-03-14	6	D3	d443	19	13	0	6	0,68	0,00	0,32	0,32	non release farm
2014	19-02-14	25-02-14	04-03-14	6	D4	d444	28	16	0	12	0,57	0,00	0,43	0,43	non release farm
2014	19-02-14	25-02-14	04-03-14	6	D5	d445	16	6	0	10	0,38	0,00	0,63	0,63	non release farm
2014	19-02-14	25-02-14	04-03-14	6	D6	d446	36	23	0	13	0,64	0,00	0,36	0,36	non release farm
2014	19-02-14	25-02-14	04-03-14	6	D7	d447	24	14	0	10	0,58	0,00	0,42	0,42	non release farm
2014	19-02-14	25-02-14	04-03-14	6	D8	d448	27	20	0	7	0,74	0,00	0,26	0,26	non release farm
2014	19-02-14	25-02-14	04-03-14	6	D9	d449	26	17	0	9	0,65	0,00	0,35	0,35	non release farm
2014	19-02-14	25-02-14	04-03-14	6	D10	d450	24	18	0	6	0,75	0,00	0,25	0,25	non release farm
2014	04-03-14	09-03-14	16-03-14	7	D1	d451	23	13	0	10	0,57	0,00	0,43	0,43	non release farm
2014	04-03-14	09-03-14	16-03-14	7	D2	d452	21	15	0	6	0,71	0,00	0,29	0,29	non release farm
2014	04-03-14	09-03-14	16-03-14	7	D3	d453	19	14	0	5	0,74	0,00	0,26	0,26	non release farm
2014	04-03-14	09-03-14	16-03-14	7	D4	d454	20	10	0	10	0,50	0,00	0,50	0,50	non release farm
2014	04-03-14	09-03-14	16-03-14	7	D5	d455	16	9	0	7	0,56	0,00	0,44	0,44	non release farm
2014	04-03-14	09-03-14	16-03-14	7	D6	d456	25	12	0	13	0,48	0,00	0,52	0,52	non release farm
2014	04-03-14	09-03-14	16-03-14	7	D7	d457	22	10	0	12	0,45	0,00	0,55	0,55	non release farm
2014	04-03-14	09-03-14	16-03-14	7	D8	d458	16	10	0	6	0,63	0,00	0,38	0,38	non release farm
2014	04-03-14	09-03-14	16-03-14	7	D9	d459	28	11	0	17	0,39	0,00	0,61	0,61	non release farm
2014	04-03-14	09-03-14	16-03-14	7	D10	d460	14	9	0	5	0,64	0,00	0,36	0,36	non release farm
2014	16-03-14	21-03-14	28-03-14	8	D1	d461	30	21	0	9	0,70	0,00	0,30	0,30	non release farm
2014	16-03-14	21-03-14	28-03-14	8	D2	d462	29	22	0	7	0,76	0,00	0,24	0,24	non release farm
2014	16-03-14	21-03-14	28-03-14	8	D3	d463	19	11	0	6	0,58	0,00	0,32	0,32	non release farm
2014	16-03-14	21-03-14	28-03-14	8	D4	d464	21	16	0	5	0,76	0,00	0,24	0,24	non release farm
2014	16-03-14	21-03-14	28-03-14	8	D5	d465	31	21	0	10	0,68	0,00	0,32	0,32	non release farm
2014	16-03-14	21-03-14	28-03-14	8	D6	d466	22	17	0	5	0,77	0,00	0,23	0,23	non release farm
2014	16-03-14	21-03-14	28-03-14	8	D7	d467	18	11	0	7	0,61	0,00	0,39	0,39	non release farm
2014	16-03-14	21-03-14	28-03-14	8	D8	d468	20	12	0	8	0,60	0,00	0,40	0,40	non release farm
2014	16-03-14	21-03-14	28-03-14	8	D9	d469	14	7	0	7	0,50	0,00	0,50	0,50	non release farm
2014	16-03-14	21-03-14	28-03-14	8	D10	d470	22	14	0	8	0,64	0,00	0,36	0,36	non release farm
2014	28-03-14	04-04-14	11-04-14	9	D1	d471	32	21	0	11	0,66	0,00	0,34	0,34	non release farm
2014	28-03-14	04-04-14	11-04-14	9	D2	d472	21	12	0	9	0,57	0,00	0,43	0,43	non release farm
2014	28-03-14	04-04-14	11-04-14	9	D3	d473	19	13	0	6	0,68	0,00	0,32	0,32	non release farm
2014	28-03-14	04-04-14	11-04-14	9	D4	d474	29	18	0	11	0,62	0,00	0,38	0,38	non release farm
2014	28-03-14	04-04-14	11-04-14	9	D5	d475	24	17	0	7	0,71	0,00	0,29	0,29	non release farm
2014	28-03-14	04-04-14	11-04-14	9	D6	d476	44	26	0	18	0,59	0,00	0,41	0,41	non release farm
2014	28-03-14	04-04-14	11-04-14	9	D7	d477	18	9	0	9	0,50	0,00	0,50	0,50	non release farm
2014	28-03-14	04-04-14	11-04-14	9	D8	d478	22	14	0	8	0,64	0,00	0,36	0,36	non release farm

2014	28-03-14	04-04-14	11-04-14	9	D9	d479	17	12	0	5	0,71	0,00	0,29	0,29	non release farm
2014	28-03-14	04-04-14	11-04-14	9	D10	d480	14	9	0	5	0,64	0,00	0,36	0,36	non release farm
2014	11-04-14	16-04-14	23-04-14	10	D1	d481	12	8	0	4	0,67	0,00	0,33	0,33	non release farm
2014	11-04-14	16-04-14	23-04-14	10	D2	d482	20	11	0	9	0,55	0,00	0,45	0,45	non release farm
2014	11-04-14	16-04-14	23-04-14	10	D3	d483	24	7	0	17	0,29	0,00	0,71	0,71	non release farm
2014	11-04-14	16-04-14	23-04-14	10	D4	d484	18	12	0	6	0,67	0,00	0,33	0,33	non release farm
2014	11-04-14	16-04-14	23-04-14	10	D5	d485	16	9	0	7	0,56	0,00	0,44	0,44	non release farm
2014	11-04-14	16-04-14	23-04-14	10	D6	d486	21	15	0	6	0,71	0,00	0,29	0,29	non release farm
2014	11-04-14	16-04-14	23-04-14	10	D7	d487	34	14	0	20	0,41	0,00	0,59	0,59	non release farm
2014	11-04-14	16-04-14	23-04-14	10	D8	d488	22	14	0	8	0,64	0,00	0,36	0,36	non release farm
2014	11-04-14	16-04-14	23-04-14	10	D9	d489	19	6	0	13	0,32	0,00	0,68	0,68	non release farm
2014	11-04-14	16-04-14	23-04-14	10	D10	d490	28	21	0	7	0,75	0,00	0,25	0,25	non release farm
2014	23-04-14	28-04-14	05-05-14	11	D1	d491	16	11	0	5	0,69	0,00	0,31	0,31	non release farm
2014	23-04-14	28-04-14	05-05-14	11	D2	d492	21	14	0	7	0,67	0,00	0,33	0,33	non release farm
2014	23-04-14	28-04-14	05-05-14	11	D3	d493	17	6	0	11	0,35	0,00	0,65	0,65	non release farm
2014	23-04-14	28-04-14	05-05-14	11	D4	d494	14	10	0	4	0,71	0,00	0,29	0,29	non release farm
2014	23-04-14	28-04-14	05-05-14	11	D5	d495	12	8	0	4	0,67	0,00	0,33	0,33	non release farm
2014	23-04-14	28-04-14	05-05-14	11	D6	d496	21	14	0	7	0,67	0,00	0,33	0,33	non release farm
2014	23-04-14	28-04-14	05-05-14	11	D7	d497	14	4	0	10	0,29	0,00	0,71	0,71	non release farm
2014	23-04-14	28-04-14	05-05-14	11	D8	d498	12	4	0	8	0,33	0,00	0,67	0,67	non release farm
2014	23-04-14	28-04-14	05-05-14	11	D9	d499	20	14	0	6	0,70	0,00	0,30	0,30	non release farm
2014	23-04-14	28-04-14	05-05-14	11	D10	d500	22	5	0	17	0,23	0,00	0,77	0,77	non release farm
2014	05-05-14	10-05-14	17-05-14	12	D1	d501	21	13	0	8	0,62	0,00	0,38	0,38	non release farm
2014	05-05-14	10-05-14	17-05-14	12	D2	d502	20	11	0	9	0,55	0,00	0,45	0,45	non release farm
2014	05-05-14	10-05-14	17-05-14	12	D3	d503	16	9	0	7	0,56	0,00	0,44	0,44	non release farm
2014	05-05-14	10-05-14	17-05-14	12	D4	d504	12	7	0	5	0,58	0,00	0,42	0,42	non release farm
2014	05-05-14	10-05-14	17-05-14	12	D5	d505	14	7	0	7	0,50	0,00	0,50	0,50	non release farm
2014	05-05-14	10-05-14	17-05-14	12	D6	d506	28	16	0	12	0,57	0,00	0,43	0,43	non release farm
2014	05-05-14	10-05-14	17-05-14	12	D7	d507	12	1	0	11	0,08	0,00	0,92	0,92	non release farm
2014	05-05-14	10-05-14	17-05-14	12	D8	d508	10	3	0	7	0,30	0,00	0,70	0,70	non release farm
2014	05-05-14	10-05-14	17-05-14	12	D9	d509	14	9	0	5	0,64	0,00	0,36	0,36	non release farm
2014	05-05-14	10-05-14	17-05-14	12	D10	d510	16	10	0	6	0,63	0,00	0,38	0,38	non release farm
2014	17-05-14	23-05-14	30-05-14	13	D1	d511	20	11	0	9	0,55	0,00	0,45	0,45	non release farm
2014	17-05-14	23-05-14	30-05-14	13	D2	d512	18	7	0	11	0,39	0,00	0,61	0,61	non release farm
2014	17-05-14	23-05-14	30-05-14	13	D3	d513	14	5	0	9	0,36	0,00	0,64	0,64	non release farm
2014	17-05-14	23-05-14	30-05-14	13	D4	d514	11	5	0	6	0,45	0,00	0,55	0,55	non release farm
2014	17-05-14	23-05-14	30-05-14	13	D5	d515	17	12	0	5	0,71	0,00	0,29	0,29	non release farm
2014	17-05-14	23-05-14	30-05-14	13	D6	d516	14	11	0	3	0,79	0,00	0,21	0,21	non release farm
2014	17-05-14	23-05-14	30-05-14	13	D7	d517	12	4	0	8	0,33	0,00	0,67	0,67	non release farm
2014	17-05-14	23-05-14	30-05-14	13	D8	d518	16	10	0	6	0,63	0,00	0,38	0,38	non release farm

2014	17-05-14	23-05-14	30-05-14	13	D9	d519	19	6	0	13	0,32	0,00	0,68	0,68	non release farm
2014	17-05-14	23-05-14	30-05-14	13	D10	d520	14	4	0	10	0,29	0,00	0,71	0,71	non release farm
