# Supplementary material for: Medfly Population Suppression through Augmentative Release of an Introduced Parasitoid in an Irrigated Multi-Fruit Orchard of Central–Western Argentina
Source: Insects. 2023 Apr 16;14(4):387. doi: 10.3390/insects14040387 (PMC10140834; doi:10.3390/insects14040387)
Supplement: Supplementary file 1 [file insects-14-00387-s001.zip › File S9_medfly R script.pdf]

```
# R script
# Suárez et al.
# Medfly population suppression through augmentative release of an introduced parasitoid in
an irrigated multi-fruit orchard of central-western Argentina**
# INSECTS

# Mean DB
# load data
library(readxl)
# db release
xm <- read_excel("xmedfly.xlsx", sheet=1)

# db structure
names(xm)
str(xm)
xm$season <- as.factor(xm$season)
xm$treatment <- as.factor(xm$treatment)
xm$idrelease <- as.factor(xm$idrelease)
xm$idtrial <- as.factor(xm$idtrial)
str(xm)


# full DB
fm <- read_excel("fullmedfly.xlsx", sheet=1)

# db structure
names(fm)
str(fm)
fm$season <- as.factor(fm$season)
fm$treatment <- as.factor(fm$treatment)
fm$idrelease <- as.factor(fm$idrelease)
fm$iddevice <- as.factor(fm$iddevice)
fm$idd <- as.factor(fm$idd)
str(fm)

### means and sd
library(doBy)
names(xm)
xtable <- summaryBy(pupaC.mean + adultC.mean +   adultD.mean + nopupaC.mean +
emergC.mean +     emergD.mean + ftd.mean + femrelease.mean +
xtemp.mean + xhr.mean ~ treatment + season, FUN=c(mean,sd), data=xm)

# export table - excel
write.table(xtable, file="xtable.txt", row.names=F)
head(xtable)

#### Correlograms mean DB
```

```
library(ellipse)
library(lattice)

# previous function
panel.corrgram <- function(x,y,z, at, level=0.9, label=False, ...)
{
	require("ellipse", quietly=T)
	zcol = level.colors(z, at=at,
		col.regions=colorRampPalette(c("red","white","blue")))
	for (i in seq(along=z)){
		ell <- ellipse(z[i], level=level, npoints=50,
		scale=c(.2,.2), centre=c(x[i], y[i]))
		panel.polygon(ell, col=zcol[i],
		border=zcol[i], ...)
	}
	if(label)
		panel.text(x=x, y=y, lab=100*round(z, 2),
		cex=0.8,col=ifelse(z<0, "white", "black"))
}

# correlations
names(xm)
# subset
levels(xm$treatment)
xmnr <- subset(xm, treatment=="non release farm")
# Cc variables no release
xm1 <-xmnr[, c(5,6,8,9)]
colnames(xm1)<-c("recovered puparia","emerged medfly adults",
"unemerged puparia", "medfly adult emergence")
cord<-cor(xm1, use="pairwise.complete.obs")
oldplot <- options(digits=1)
cord
options(oldplot)

# figure
print(levelplot(cord, at=do.breaks(c(-1.01,1.01), 20),
	xlab=NULL, ylab=NULL, colorkey=list(space="top",
	col=colorRampPalette(c("red","white","blue"))), scales=list(x=list(rot=90)),
	panel=panel.corrgram,
	label=T))

# correlation table
library(rstatix)
names(xm1)
c1 <- xm1 %>% cor_test("medfly adult emergence")
write.table(c1, file="cor1.txt",
	row.names=F)
c1
```

```
#### Correlograms emergence - mean DB
# correlations
names(xm)
# subset
levels(xm$treatment)
xmnr <- subset(xm, treatment=="non release farm")
# Cc variables no release
xm2 <-xmnr[, c(9,23,25,26,28)]
colnames(xm2)<-c("medfly adult emergence","FTD",
"air temperature", "air humidity", "accumulated rainfall")
cord<-cor(xm2, use="pairwise.complete.obs")
oldplot <- options(digits=1)
cord
options(oldplot)

# figure
print(levelplot(cord, at=do.breaks(c(-1.01,1.01), 20),
        xlab=NULL, ylab=NULL, colorkey=list(space="top",
        col=colorRampPalette(c("red","white","blue"))), scales=list(x=list(rot=90)),
        panel=panel.corrgram,
        label=T))

# Correlation table
library(rstatix)
names(xm2)
c2 <- xm2 %>% cor_test("medfly adult emergence")
write.table(c2, file="cor2.txt",
        row.names=F)
c2

#### Correlograms Da
# correlations
names(xm)
# subset
levels(xm$treatment)
xmnr <- subset(xm, treatment=="release farm")
# variables no release
xm3 <-xmnr[, c(10,24,25,26,28)]
names(xm3)
colnames(xm3)<-c("emerged female parasitoids", "released female parasitoids",
"air temperature", "air humidity", "accumulated rainfall")
cord<-cor(xm3, use="pairwise.complete.obs")
oldplot <- options(digits=1)
cord
options(oldplot)

# figure
```

```
print(levelplot(cord, at=do.breaks(c(-1.01,1.01), 20),
          xlab=NULL, ylab=NULL, colorkey=list(space="top",
          col=colorRampPalette(c("red","white","blue"))), scales=list(x=list(rot=90)),
          panel=panel.corrgram,
          label=T))

# Correlation table Da
library(rstatix)
names(xm3)
c3 <- xm3 %>% cor_test("emerged female parasitoids")
write.table(c3, file="cor3.txt",
          row.names=F)
c3

# gls
library(car)
library(lattice)
library(latticeExtra)
library(nlme)
library(nlstools)
library(visreg)

# gls emergence Cc temperature
# nesting
names(xm)
nxm <- groupedData(emergC.mean ~ season|idrelease,
               data= xm)
names(nxm)
g1 <- gls(emergC.mean ~ treatment * season +
          xtemp.mean , data=nxm,
        corr=corARMA(p=1,q=1),
        weights = NULL, method="REML")
summary(g1)
anova(g1)

# figure
library(visreg)
op <- par(mfrow=c(2,2), mar=c(4,4,2,2),
        mgp=c(2.3,1,0))
             visreg(g1,"treatment",
              line=list(col="forestgreen", lwd=3),
              points=list(col="blue", cex=1.1),
              cex.lab=1.3,ylab="medfly emergence",xlab="treatment")
             visreg(g1,"season",
              line=list(col="forestgreen", lwd=3),
              points=list(col="blue", cex=1.1),
              cex.lab=1.3, ylab="", xlab="season")
             visreg(g1,"xtemp.mean",
```

```
                    line=list(col="forestgreen", lwd=3),
                    points=list(col="blue", cex=1.1),
                    cex.lab=1.3,ylab="medfly emergence",xlab="air temperature")
par(op)
layout(1)

# gls emergence Cc within releasing period
# nesting
names(fm)
fm$emergC <- fm$`medfly emergence`
names(fm)
nfm <- groupedData(emergC ~          idrelease|iddevice, data= fm)
names(nfm)
g2 <- gls(emergC ~ treatment * season * idrelease
          , data=nfm,
         corr=corARMA(p=1,q=1),
         weights = NULL, method="REML")
summary(g2)
anova(g2)

# figure
library(visreg)
op <- par(mfrow=c(2,2), mar=c(4,4,2,2),
         mgp=c(2.3,1,0))
                   visreg(g2,"treatment",
                    line=list(col="forestgreen", lwd=3),
                    points=list(col="blue", cex=1.1),
                    cex.lab=1.3,ylab="emergence",xlab="treatment")
                   visreg(g2,"season",
                    line=list(col="forestgreen", lwd=3),
                    points=list(col="blue", cex=1.1),
                    cex.lab=1.3, ylab="", xlab="season")
                   visreg(g2,"idrelease",
                    line=list(col="forestgreen", lwd=3),
                    points=list(col="blue", cex=1.1),
                    cex.lab=1.3,ylab="emergence",xlab="releasing period")
par(op)
layout(1)

library(visreg)
op <- par(mfrow=c(2,2), mar=c(4,4,2,2),
         mgp=c(2.3,1,0))
                   visreg(g2,"treatment", by="season",
                    line=list(col="forestgreen", lwd=3),
                    points=list(col="blue", cex=1.1),
                    cex.lab=1.3,ylab="emergence",xlab="treatment")
                   visreg(g2,"idrelease",by="treatment",
                    line=list(col="forestgreen", lwd=3),
```

```
                    points=list(col="blue", cex=1.1),
                    cex.lab=1.3,ylab="emergence",xlab="releasing period")
par(op)
layout(1)

# Selected model***
# nesting
names(fm)
nfm <- groupedData(emergC ~
          idrelease|iddevice, data= fm)
names(nfm)
g3 <- gls(emergC ~ treatment * idrelease + season, data=nfm,
        corr=corARMA(p=1,q=1),
        weights = NULL, method="REML")
summary(g3)
anova(g3)

# figures
visreg(g3,"treatment", by="idrelease",
                    line=list(col="forestgreen", lwd=3),
                    points=list(col="blue", cex=1.1),
                    cex.lab=1.3,ylab="medfly emergence",xlab="treatment")

visreg(g3,"idrelease",by="treatment",
                    line=list(col="forestgreen", lwd=3),
                    points=list(col="blue", cex=1.1),
                    cex.lab=1.3,ylab="medfly emergence",xlab="releasing period")

# mortality - nesting
names(fm)
fm$mortC <- fm$`medfly mortality`
nfm2 <- groupedData(mortC ~
          idrelease|iddevice, data= fm)
names(nfm2)
g4 <- gls(mortC ~ treatment * idrelease + season
         , data=nfm2,
        corr=corARMA(p=1,q=1),
        weights = NULL, method="REML")
summary(g4)
anova(g4)

# figures
library(visreg)
visreg(g4,"treatment", by="idrelease",
                    line=list(col="forestgreen", lwd=3),
                    points=list(col="blue", cex=1.1),
                    cex.lab=1.3,ylab="mortality",xlab="treatment")
```

```
visreg(g4,"idrelease",by="treatment",
                    line=list(col="forestgreen", lwd=3),
                    points=list(col="blue", cex=1.1),
                    cex.lab=1.3,ylab="mortality",xlab="releasing period")

# Descriptive figures
# tidy db
library(tidyr)
names(fm)

numberfm <- fm %>%
  pivot_longer("puparia":"parasitoids", names_to = "variable", values_to = "individuals")

indexfm <- fm %>%
  pivot_longer("medfly emergence":"medfly mortality", names_to = "variable", values_to =
"individuals")

# medfly by year and release date - individuals ***
library(ggplot2)
names(numberfm)
ggplot(data=numberfm, aes(x=idrelease, y= individuals, fill= treatment))+
  geom_boxplot()+
  theme_bw()+
  facet_grid(season ~ variable) +
  theme(axis.text.x = element_text(angle = 0, vjust = 0.5, hjust=1)) +
  theme(legend.position="top") +
  labs(title="", subtitle="", caption="", x="releasing period", y="individuals")

# medfly by year and release date - proportions ***
library(ggplot2)
names(indexfm)
ggplot(data=indexfm, aes(x=idrelease, y= individuals, fill= treatment))+
  geom_boxplot()+
  theme_bw()+
  facet_grid(season ~ variable) +
  theme(axis.text.x = element_text(angle = 0, vjust = 0.5, hjust=1)) +
  theme(legend.position="top") +
  labs(title="", subtitle="", caption="", x="releasing period", y="proportion")

# end of script
```
