# Supplementary figures and images for: Medfly Population Suppression through Augmentative Release of an Introduced Parasitoid in an Irrigated Multi-Fruit Orchard of Central–Western Argentina
Source: Insects. 2023 Apr 16;14(4):387. doi: 10.3390/insects14040387 (PMC10140834; doi:10.3390/insects14040387)

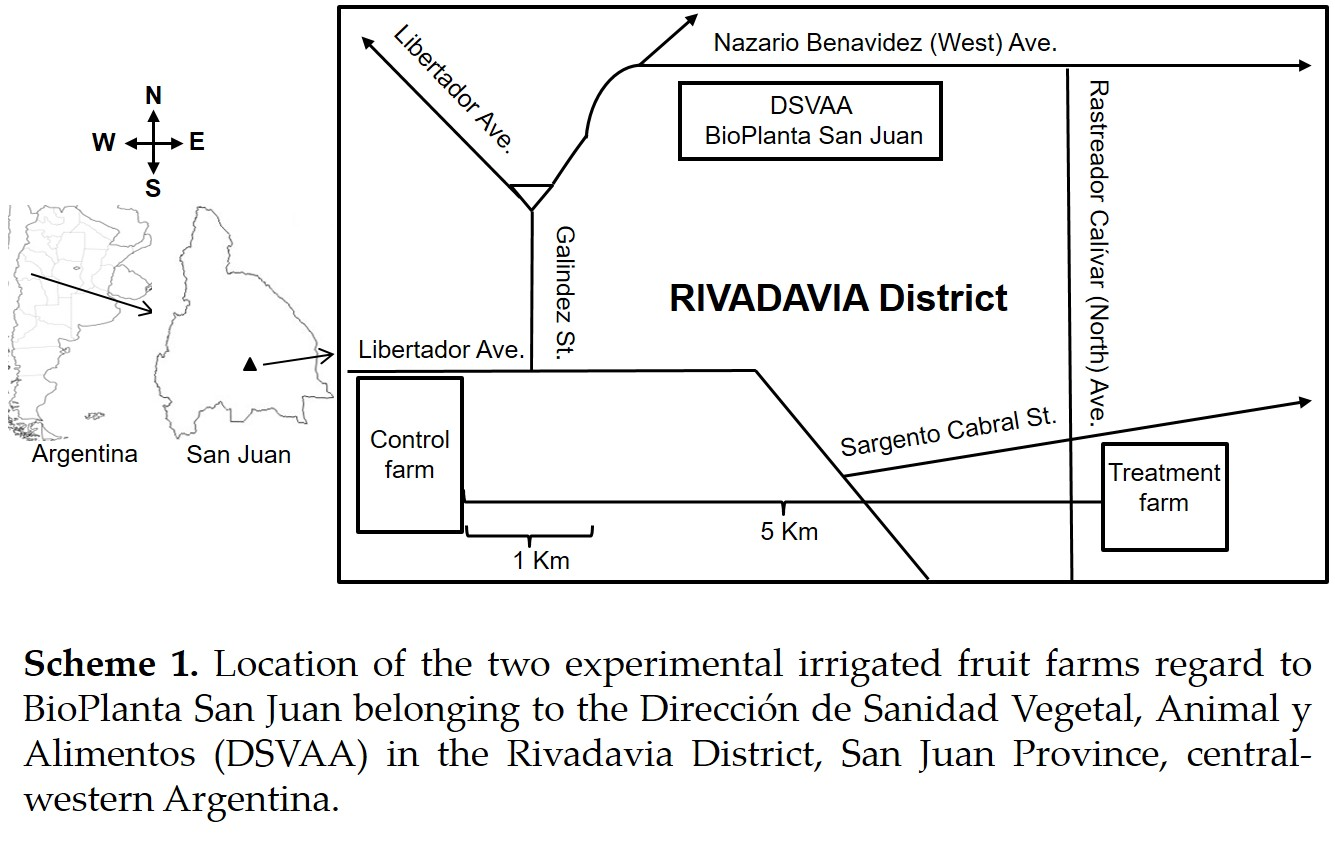

Supplement: Supplementary file 1 [file insects-14-00387-s001.zip › File S1_Scheme 1.tiff]

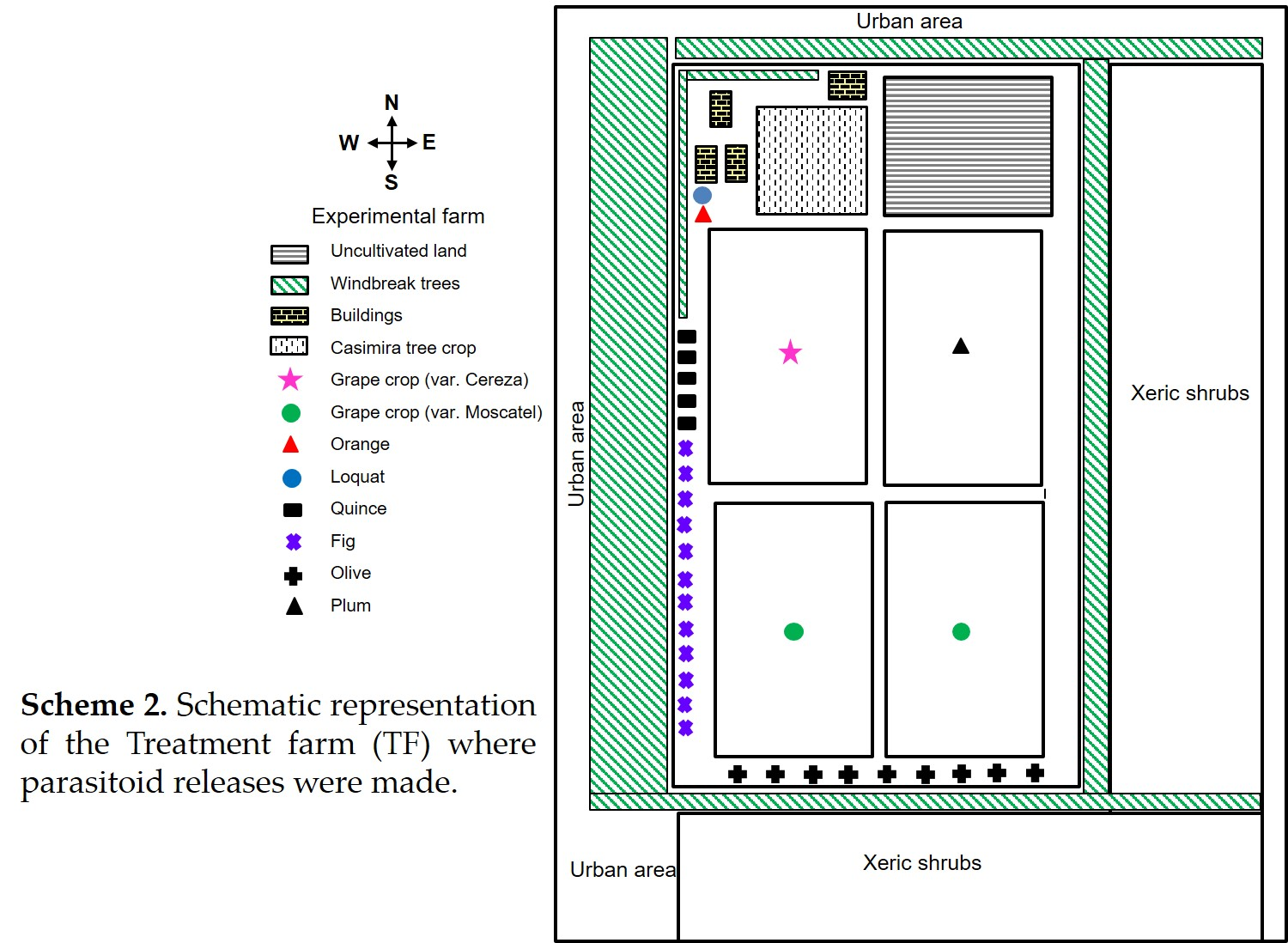

Supplement: Supplementary file 1 [file insects-14-00387-s001.zip › File S2_Scheme 2.tiff]

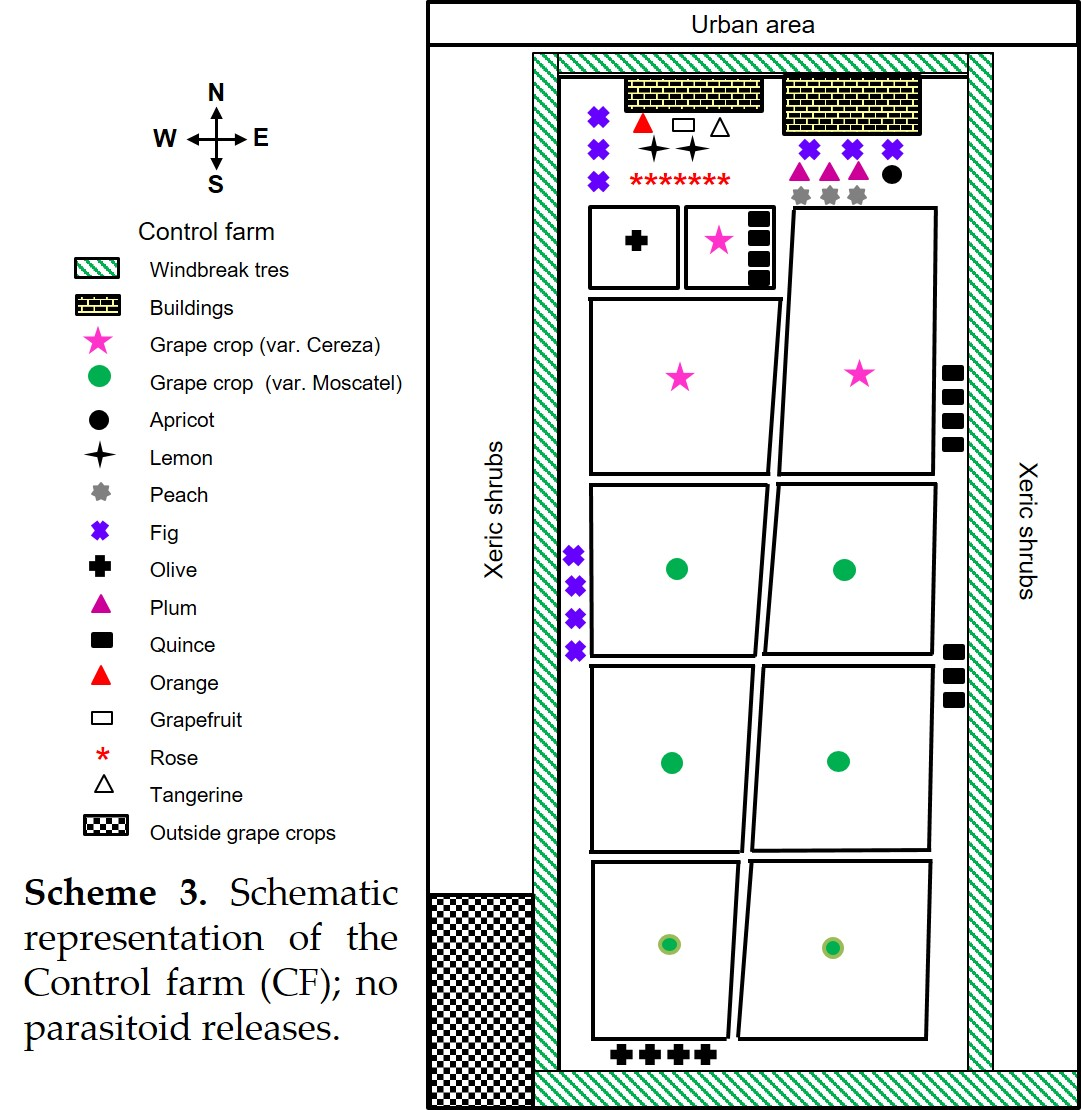

Supplement: Supplementary file 1 [file insects-14-00387-s001.zip › File S3_Scheme 3.tiff]

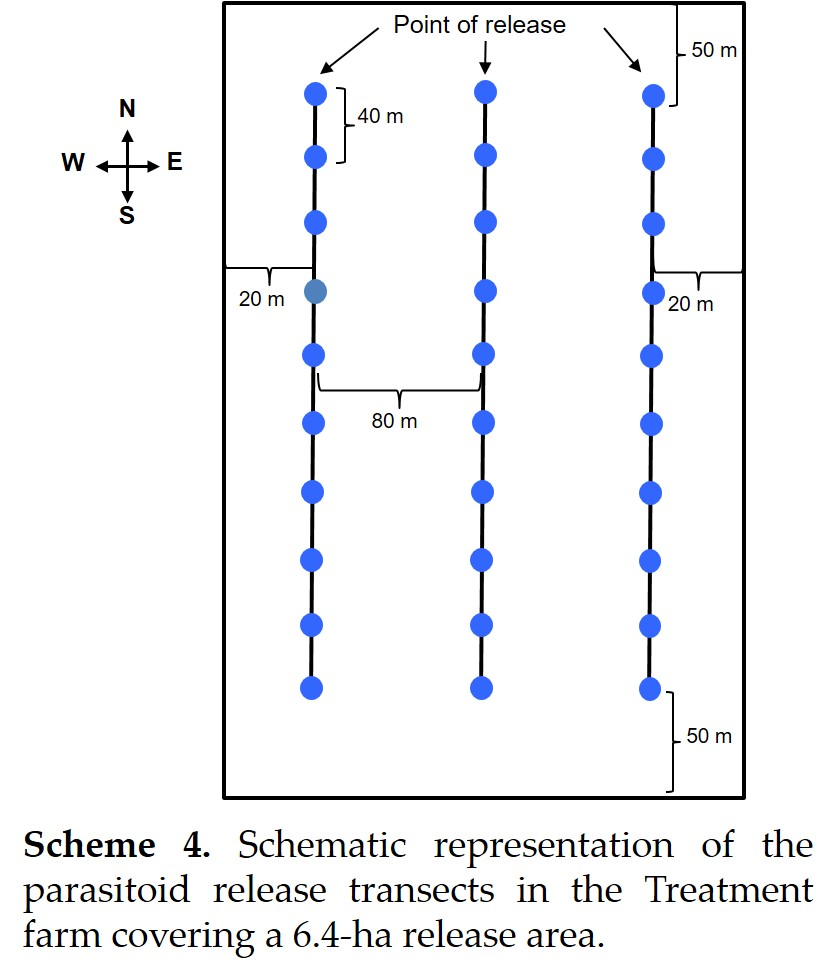

Supplement: Supplementary file 1 [file insects-14-00387-s001.zip › File S4_Scheme 4.tiff]

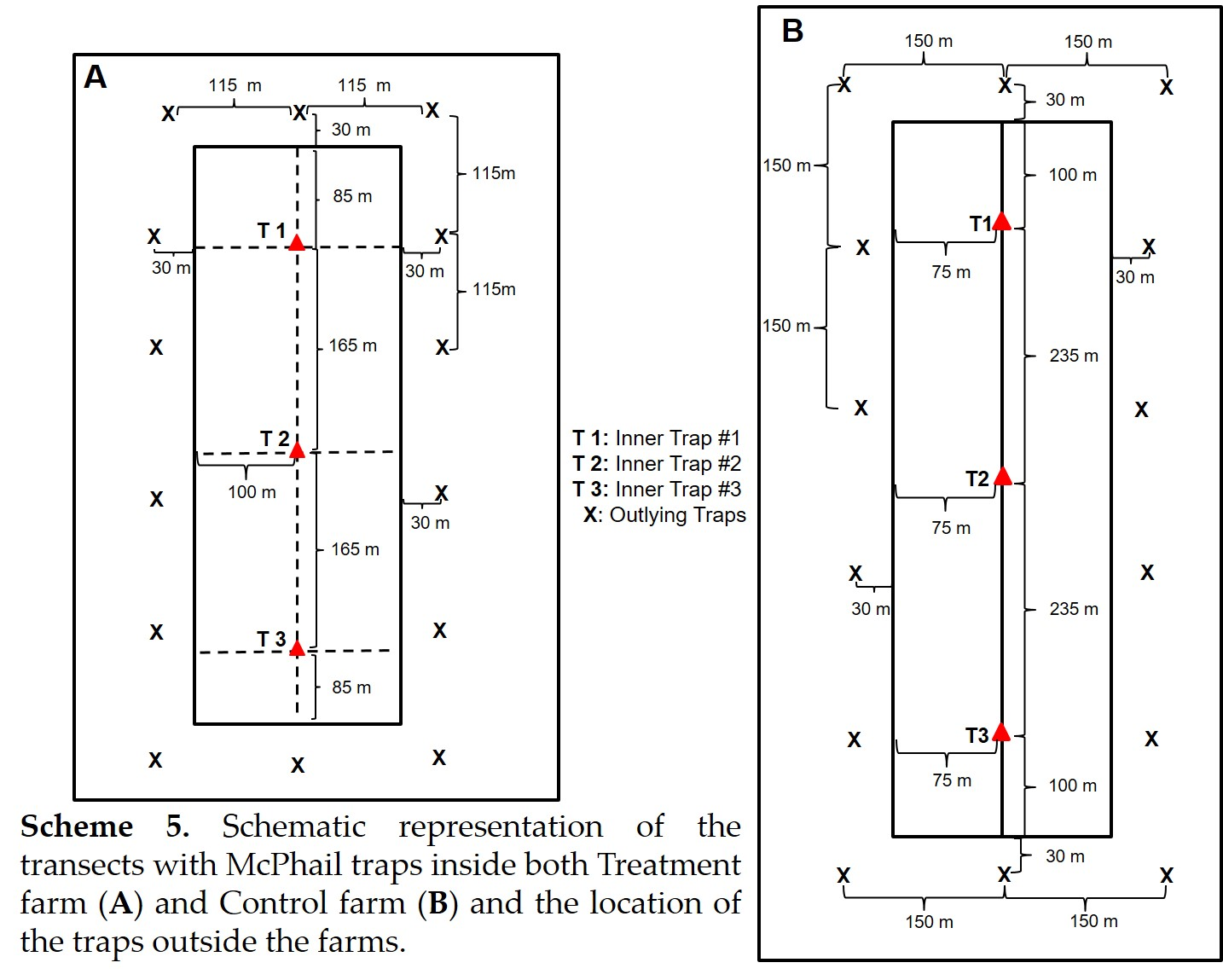

Supplement: Supplementary file 1 [file insects-14-00387-s001.zip › File S5_Scheme 5.tiff]

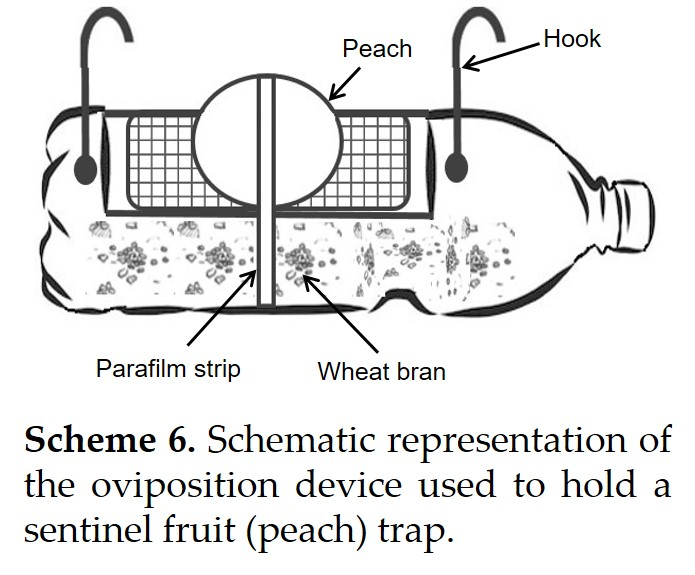

Supplement: Supplementary file 1 [file insects-14-00387-s001.zip › File S6_Scheme 6.tiff]

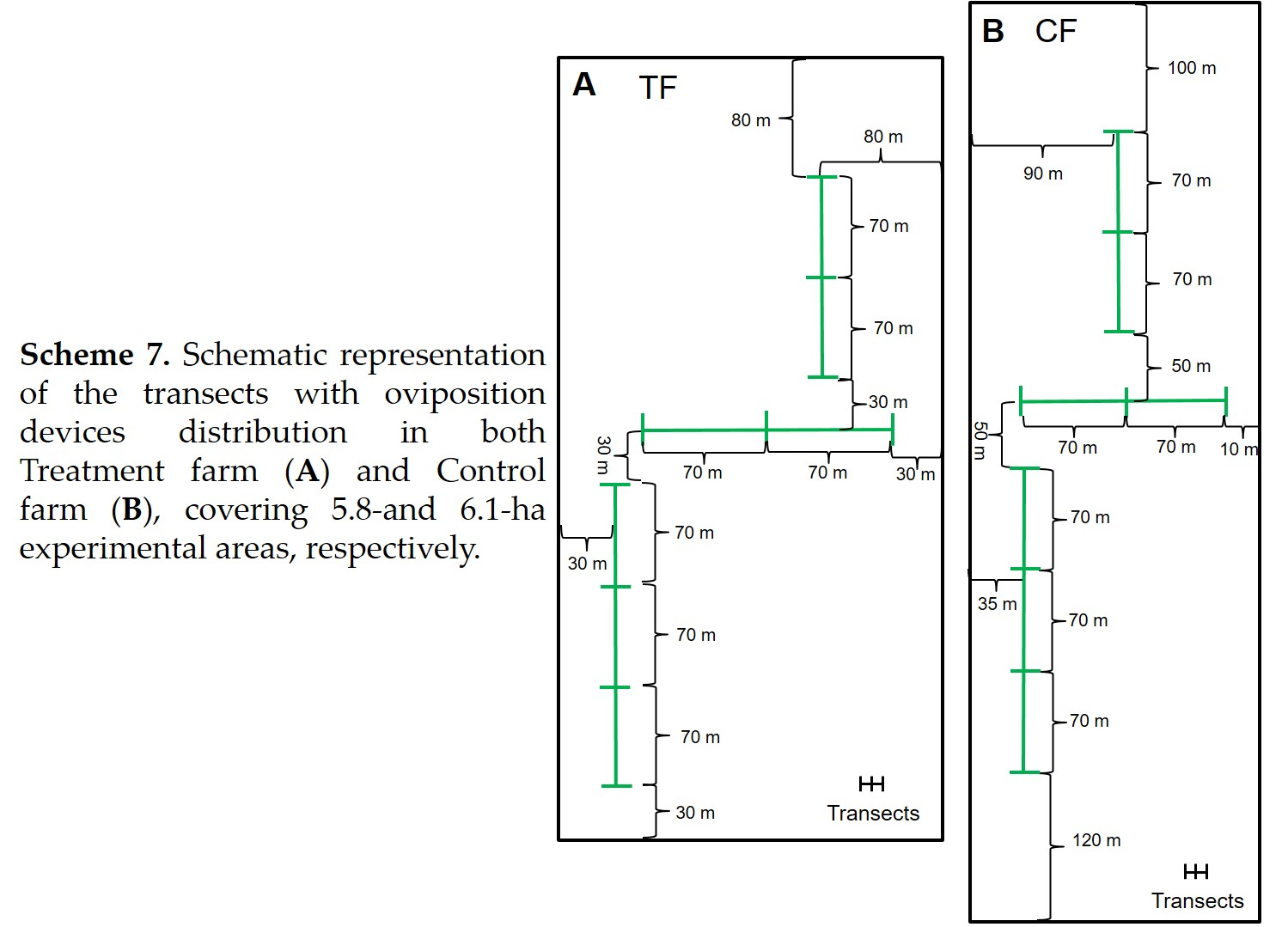

Supplement: Supplementary file 1 [file insects-14-00387-s001.zip › File S7_Scheme 7.tiff]
